# ¹⁸F-FDG PET radiomics and machine learning for virtual biopsy and treatment decisions in lymphoma: a multicenter study

**DOI:** 10.1007/s13246-025-01675-2

**Published:** 2025-11-20

**Authors:** Setareh Hasanabadi, Seyed Mahmud Reza Aghamiri, Ahmad Ali Abin, Mehrdad Bakhshayesh Karam, Habibeh Vosoughi, Farshad Emami, Elham Askari, Sharareh Seifi, Atosa Dorudinia, Hossein Arabi, Habib Zaidi

**Affiliations:** 1 Department of Medical Radiation Engineering, Shahid Beheshti University, Tehran, Iran; 2 Faculty of Computer Science and Engineering, Shahid Beheshti University, Tehran, Iran; 3 National Research Institute of Tuberculosis and Lung Diseases, Shahid Beheshti University of Medical Sciences, Tehran, Iran; 4 Nuclear Medicine Department, Masih Daneshvari Hospital, Tehran, Iran; 5 Research Center for Nuclear Medicine, Tehran University of Medical Sciences, Tehran, Iran; 6 Razavi Cancer Research Center, Razavi Hospital, Imam Reza International University, Mashhad, Iran; 7 Vanak pathobiology lab, Vanak, Tehran, Iran; 8 Division of Nuclear Medicine & Molecular Imaging, Geneva University Hospital, CH-1211 Geneva, Switzerland; 9 Department of Nuclear Medicine and Molecular Imaging, University of Groningen, Groningen, Netherlands; 10 Department of Nuclear Medicine, University of Southern Denmark, Odense, Denmark; 11 University Research and Innovation Center, Óbuda University, Budapest, Hungary

**Keywords:** PET, Lymphoma, Radiomics, Machine learning, Multi-center study

## Abstract

This study investigated the potential of combining baseline ^18^F-FDG PET tumor-to-liver ratio radiomics with demographic data, using machine learning, to classify lymphoma subtypes and differentiate between candidates for ABVD and R-CHOP therapy. Additionally, we assessed whether nodal radiomics alone is sufficient for treatment and subtype classification. We conducted a multi-center study involving 241 lymphoma patients, including 125 with Non-Hodgkin lymphoma (NHL) and 116 with Hodgkin lymphoma. Among these, 94 had high-grade NHL, whereas 110 had classical Hodgkin lymphoma. We utilized 107 radiomic features, along with demographic data, such as age, stage, gender, and weight, to develop predictive models for classifying lymphoma subtypes and selecting treatment regimens (ABVD vs. R-CHOP). Data harmonization was performed using ComBat, feature selection was done with SelectKBest, and three machine learning models (Logistic Regression, Random Forest, and XGBoost) were trained with hyperparameter tuning, followed by external validation. For the best model in each classifier on the external test, adding extra-nodal radiomic features improved performance for certain lymphoma subtypes. For NHL vs. HL, accuracy increased from 0.807–0.819, whereas NHL precision rose from 0.837–0.875. High-grade NHL precision improved notably from 0.821–0.962. In treatment classification, extra-nodal features boosted accuracy for R-CHOP from 0.783–0.839 and increased F1-scores for both R-CHOP and ABVD. This study demonstrated the promise of PET radiomics combined with demographic features for lymphoma classification and treatment decision-making. Overall, extra-nodal features enhanced high-grade NHL and treatment classification but had minimal impact on other lymphoma subtypes.

**Supplementary Information:**

The online version contains supplementary material available at 10.1007/s13246-025-01675-2.

## Introduction

Lymphoma, making up 5% of all cancers, is the most common hematological malignancy. It is classified into Hodgkin lymphoma (10%) and Non-Hodgkin lymphoma (NHL) (90%). These cancers result from abnormal lymphocyte growth, with an overall survival rate of around 72% [1, 2]. However, outcomes vary significantly by subtype [3]. Thus, an accurate and timely diagnosis is essential for optimizing treatment strategies and improving patient outcomes.

Given the importance of accurate and timely diagnosis for optimizing treatment strategies, tissue biopsy remains the gold standard for diagnosing most hematopoietic and lymphoid malignancies [4]. However, as an invasive procedure, it has limitations such as insufficient sample size, subjective interpretation, procedural risks, and spatial constraints [5], highlighting the need for alternative methods. Furthermore, lymphomas can transform into more aggressive forms [3] at any time after diagnosis, requiring repeated biopsies to confirm the transformation. This complicates diagnosis and underscores the need for non-invasive methods [6].

Considering the challenges associated with repeated biopsies and the importance of finding non-invasive diagnostic options, ^18^F-fluorodeoxyglucose PET/CT (^18^F-FDG PET/CT) has become a widely adopted method for initial staging, restaging, treatment assessment, and follow-up of lymphoma patients. It provides three-dimensional imaging that facilitates effective monitoring of disease progression, despite its limitations, such as false positives and negatives [7–9].

Despite its utility in managing lymphoma, this modality cannot be directly used to estimate lymphoma subtypes. However, it can serve as a foundation for developing non-invasive tools for virtual biopsy. Since relying solely on imaging is not feasible, we propose combining radiomic features extracted from PET imaging with a machine learning model to achieve more accurate and comprehensive results. Recent advancements in radiomic techniques, with or without the integration of machine learning, utilize extensive quantitative data from PET/CT imaging and have shown promise in differentiating lymphoma from other malignancies [10–17]. However, when it comes to differentiating lymphoma subtypes, existing studies are limited and have notable constraints [18–21]. Most of these studies are conducted in single-center settings, often using data from a single geographic region, which can introduce biases and limit their applicability to diverse patient populations. Moreover, the lack of diversity among the lymphoma subtypes studied restricts the generalizability of the findings. Additionally, studies such as the one conducted by de Jesus et al. [19], which focus only on tumors with high standardized uptake value (SUV_max_), potentially biasing their results towards high SUV cases. For the first time, we employed a multi-faceted approach to lesion selection, moving beyond the conventional focus on the hottest or most bulky lesion alone.

In this study, our primary objective was to develop a non-invasive diagnostic tool as an alternative to traditional lymphoma biopsy by integrating tumor-to-liver ratio (TLR) PET radiomics, patient demographic data, and machine learning, aiming for an efficient and accurate solution. This approach, referred to as a “virtual biopsy”, serves as a non-invasive surrogate for tissue biopsy. It can reduce the need for multiple invasive procedures and is particularly useful when lesions are difficult to access or when biopsy poses a risk to the patient, while histopathology remains the gold standard.

To fill gaps from previous studies, we implemented a multi-center framework involving patients from two centers with significant geographical and ethnic diversity. Our secondary goal was to evaluate whether radiomics combined with patient demographic features and machine learning could classify patients, like an oncologist, into appropriate first-line treatment regimens. Given that lymphoma is a systemic disease that can affect multiple lymph nodes and spread to various parts of the body, this work presents a major challenge in determining which tumor to consider for segmentation, feature extraction, and radiomic analysis. For the first time, we aimed to determine whether nodal radiomics with demographic data alone is sufficient or if extranodal radiomics should be included for better outcomes. To the best of our knowledge, no prior study has combined TLR features with demographic data while considering both nodal and extranodal radiomics in a multi-center study involving ethnically diverse patients for lymphoma classification and therapy decision-making.

## Methods

### Patient demographics and study design

A retrospective cohort study was conducted at two centers: the main center (Masih Daneshvari Hospital, Tehran, Iran) and a secondary center (Razavi Hospital, Mashhad, Iran) from 2014 to 2024. The study was approved by the Medical Ethical Review Committee of Shahid Beheshti University of Medical Sciences, under ethical code IR.SBMU.NRITLD.REC.1402.060. Due to the non-interventional and retrospective nature of the study, informed consent was waived by the committee. Patients with an initial diagnosis of lymphoma who underwent a baseline ^18^F-FDG PET/CT scan and had histopathological confirmation were included.

There was significant geographical and ethnic diversity between patients from the two centers. At Center 2 (Razavi Hospital), over 80% of patients were of Arab descent, while the remaining patients were from the northeastern part of Iran, most of whom were also of Arab origin. In contrast, at Center 1 (Masih Daneshvari Hospital), more than 95% of the patients were of Iranian descent, primarily from the central regions of the country.

### Patient selection criteria

Patients were excluded from the study if they had incomplete imaging data. Patients who lacked histopathological confirmation were also excluded. Additionally, those who had received any treatment prior to the PET/CT scan were not included in the study. Additional exclusion criteria included negative or inconclusive diagnostic results. Individuals with suspected concurrent infections were also excluded. Moreover, patients with liver fibrosis or cirrhosis that could impair normal liver uptake were not included. Furthermore, individuals with concurrent or recent malignancies, such as breast cancer, were excluded. Patients whose PET/CT images were compromised by artifacts or poor image quality were also excluded.

### Pathological and oncological classification

One pathologist with over 15 years and another with more than 6 years of experience classified the lymphoma cases based on the WHO guidelines [22], their expertise, and pathology reports. The classification process first separated the cases into Hodgkin lymphoma and NHL lymphoma. Hodgkin lymphoma was further classified into classical Hodgkin lymphoma (CHL) and nodular lymphocyte-predominant Hodgkin lymphoma (NLPHL), while NHL lymphoma was divided into aggressive and low-grade NHL lymphomas, as well as B-cell and other types.

Additionally, one oncologist with over 14 years of experience assessed the patients and divided them into two therapeutic groups. The first group consisted of candidates for Adriamycin, Bleomycin, Vinblastine, Dacarbazine (ABVD) therapy, while the second group was for patients receiving Rituximab, Cyclophosphamide, Doxorubicin, Vincristine, Prednisone (R-CHOP) therapy. These decisions were made based on her extensive clinical experience.

### PET/CT imaging protocol

#### Center 1

PET/CT examinations were carried out on a GE Discovery 690 scanner equipped with Time-of-Flight (TOF) capability and a 64-slice CT. Whole-body scans spanned from the top of the head (vertex) down to the mid-thigh. Image reconstruction was performed using the VUE Point HD/FX technique. The average uptake period was 60 min, with individual uptake times varying between 45 and 75 min. Each PET imaging bed position was scanned for two to three minutes. PET scans maintained a slice thickness of 3.75 mm, whereas the low-dose CT scans had slice thicknesses ranging from 1.33–2.5 mm. The X-ray tube current was automatically adjusted using the Smart mAs algorithm based on patient’s weight, with settings between 50 and 150 mA. The tube voltage was set at 120 kVp, and the helical pitch factor was consistently maintained at 0.9. ^18^F-FDG PET images were corrected for scatter and attenuation using data derived from the CT scans.

#### Center 2

The Biograph 6 TrueV scanner was used for whole-body PET/CT examinations performed one hour after intravenous administration of ¹⁸F-FDG, with uptake times ranging from 50–70 min. The protocol involved injecting 0.1 mCi (3.7 MBq) of ¹⁸F-FDG per kilogram of body weight. Each PET scanning bed position was imaged for 2.2 min. The CT scan used a pitch factor of 0.55. PET scans maintained a slice thickness of 5 mm, whereas the low-dose CT scans had slice thickness of 3 mm. CT images were reconstructed using filtered backprojection (FBP). For PET images, the iterative ordered subset expectation maximization (OSEM) method was applied, using 2 iterations and 21 subsets.

### Primary image evaluation and lesion segmentation

Initially, PET/CT images were evaluated by a nuclear medicine physician with over 10 years of experience to assess the exclusion criteria. The same physician then assigned an Ann Arbor stage [23], which was later confirmed by a radiologist with up to 34 years of experience. Subsequently, lesions were delineated by the nuclear medicine physician using a semi-automated graph-based method [24], integrated as an extension for the 3D Slicer software [25].The physician then made any necessary adjustments to the lesion borders.

**Fig. 1:**
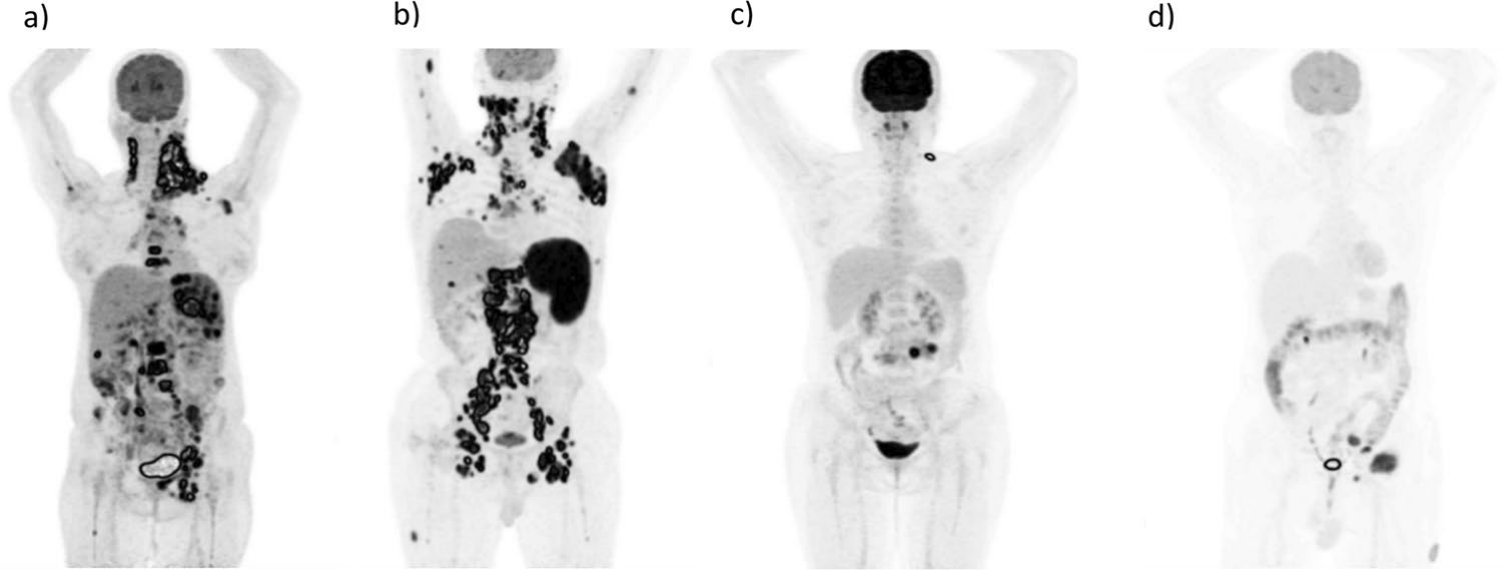
Maximum intensity projection (MIP) images of patients diagnosed with: **a** nodular sclerosis classical hodgkin lymphoma, **b** diffuse large b-cell lymphoma (DLBCL), **c** marginal zone B-Cell lymphoma, **d** T-Cell lymphoma

We segmented at least one lesion from each lymphatic and extra-lymphatic region. However, there were no limitations on the number of tumors per patient, and in some cases, more than 50 tumors were segmented from a single patient. After lesion delineation, a 3-dimensional region of interest (ROI) with three diameters was defined in the right lobe of the liver, ensuring it was free of any lesions, in accordance with the PERCIST criteria [26], to calculate TLR radiomics. As shown in Fig. 1, several types of lymphoma are illustrated, including Nodular Sclerosis Hodgkin Lymphoma, Diffuse Large B-Cell Lymphoma (DLBCL), Marginal Zone B-Cell Lymphoma, and T-Cell Lymphoma.

### Image preprocessing and feature extraction

**Fig. 2:**
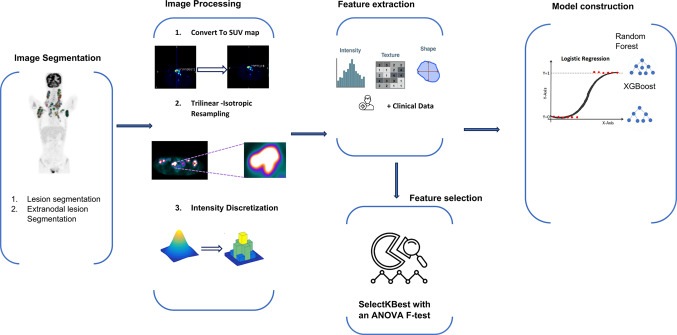
Radiomics workflow of the current study: Initial acquisition of ^18^F-FDG PET images, followed by preprocessing, segmentation of lesions, and extraction of radiomic and shape features. Machine learning classifiers were then trained using these features alongside clinical data, with the optimal model selected based on the highest AUC

Figure 2 illustrates the radiomics workflow. The first step in image analysis involved resampling the images to achieve isotropic voxel spacing. PET images were then converted into SUV maps. SUV maps were discretized, with a fixed bin size of 0.25 SUV, following the guidelines set by the Image Biomarker Standardization Initiative (IBSI) [27]. This process was carried out using the PyRadiomics extension in the 3D Slicer software [25].To calculate the tumor-to-liver ratio (TLR) of feature values, features were also extracted from the liver volume of interest (VOI) on PET images, excluding shape features. Since each patient had more than one tumor, and our approach was patient-based, we first extracted radiomic features independently for each lesion. To normalize for inter-patient variability, we calculated the tumor-to-liver ratio (TLR) by dividing each lesion feature (e.g., SUV_max_) by the corresponding feature from a lesion-free spherical liver region (3 cm diameter) in the liver. After TLR normalization, lesion-level features were aggregated to patient-level features using four metrics: mean, maximum, minimum, and median, capturing both typical and extreme lesion characteristics. This approach ensures a comprehensive and reproducible representation of multi-lesion patients.

### Feature selection and machine learning elaboration

We began by harmonizing data emanating from different centers using ComBat to minimize any center-related differences. Afterward, we split the data from Center 1 into 80% for training and 20% for internal validation through a stratified split to maintain class balance. We reserved the data from Center 2 for external testing. Next, we performed feature selection using SelectKBest with an ANOVA F-test, identifying the most relevant radiomics and clinical features that contribute to the predictive power of the model. The SelectKBest method applies the F-test to evaluate the relationship between each feature and the target variable, selecting the features with the highest scores. We examined a range of values for the parameter k, which controls the number of features to be selected, from 5–50, based on the number of available features in the dataset. This step was integrated into a machine learning pipeline, where the data was first scaled, followed by feature selection, and then the model was trained using the reduced feature set. This process allowed us to focus on the most impactful features, improving the efficiency and accuracy of the model while reducing the risk of overfitting.

To optimize the number of selected features, we applied GridSearchCV, ensuring the best performance. We then trained three machine learning models—Logistic Regression (LR), Random Forest (RF), and XGBoost (XGB)—using a pipeline that included feature scaling with StandardScaler and feature selection. For each model, we fine-tuned hyperparameters, such as the learning rate and maximum depth for XGBoost, regularization strength for LR, and the number of estimators for RF, all through GridSearchCV. To ensure model robustness, we applied a stratified 5-fold cross-validation on the training data. For logistic regression, we used the coefficients to assess the influence of each predictor. For Random Forest and XGBoost, we calculated feature importance to understand which predictors had the most influence. Finally, we validated the models using the independent test set from Center 2, evaluating their performance through AUC ROC curves, and classification report metrics including accuracy, precision, recall, and F1-score. We selected the best model based on its performance on the unseen data using AUC as the primary metric.

### Classification task overview

As indicated in Table 1, we defined five classification tasks, each with a distinct clinical purpose and patient cohort. Tasks, such as ABVD vs. R-CHOP, focus on treatment selection, while high-gradeNHL vs. HL and NHL vs. HL address diagnostic differentiation. The B-cell vs. Others task explores radiomic differentiation in cases with ambiguous pathology reports. Patient inclusion criteria, cohort composition, and task-specific distinctions are provided in Table 1 for clarity.

**Table 1:** Overview of classification tasks, their clinical motivation, patient cohorts, and distinctions from similar tasks

Classification task	Clinical motivation	Patient cohort / inclusion criteria	Cohort composition	Distinction from similar tasks
ABVD vs R-CHOP candidates	Guide therapy selection: distinguish HL patients for ABVD vs high-grade B-NHL for R-CHOP	Patients who were candidates for ABVD or R-CHOP; Burkitt lymphoma excluded due to distinct treatment (e.g., CODOX-M/IVAC)	HL (ABVD) and high-grade B-NHL (R-CHOP, excluding Burkitt)	Focuses on treatment decision (ABVD vs R-CHOP); unlike High-grade B-NHL vs HL, which is diagnostic and includes more patients
High-grade NHL vs HL	Diagnostic differentiation: distinguish aggressive B-cell NHL (e.g., DLBCL) from HL	All high-grade NHL patients (including Burkitt) and all HL patients	High-grade -NHL (DLBCL, etc.) and HL (predominantly CHL)	Diagnostic focus, includes all high-grade NHL and HL patients, regardless of treatment
High-grade NHL vs CHL	Refine HL distinction focusing on classical HL (most prevalent)	All high-grade NHL patients (including Burkitt) and all CHL patients	High-grade NHL and CHL	Subset of High-grade NHL vs HL; focuses on CHL due to its high prevalence and uniform treatment
NHL vs HL	Support early diagnostic triage; differentiate all NHL (including indolent) from HL	All NHL (indolent + aggressive) and all HL patients	NHL (indolent + high-grade) and HL (predominantly CHL)	Broader than High-grade NHL vs HL; includes indolent NHL for early diagnostic triage
B-cell vs Others	Explore radiomic differentiation of B-cell lymphomas from other types; addresses ambiguous pathology reports	All B-cell lymphomas vs HL + T/NK-cell NHL	B-cell lymphomas (mainly high-grade NHL) vs HL (predominantly CHL) + ~ 15 T/NK-cell NHL	Distinct from others; focuses on identifying B-cell vs non-B-cell lymphomas for ambiguous pathology cases

ABVD vs. R-CHOP distinguishes HL patients from high-grade B-NHL receiving R-CHOP, excluding Burkitt lymphoma. High-grade NHL vs. HL differentiates aggressive B-cell NHL from HL. High-grade-NHL vs. CHL focuses on classical HL (CHL). NHL vs. HL separates all NHL subtypes from HL. B-cell vs. Others distinguishes B-cell lymphomas from HL and T/NK-cell NHL, addressing ambiguous pathology reports. Abbreviations: HL–Hodgkin lymphoma; CHL–classical HL; NHL–non-Hodgkin lymphoma; B-NHL–B-cell NHL; DLBCL–diffuse large B-cell lymphoma; T/NK-cell NHL–T/NK-cell lymphoma

## Results

### Patient demographics

This study involved 241 patients, including 159 from Center 1 and 82 from Center 2, all diagnosed with different types of lymphoma. Of these, 125 had NHL, and 116 had Hodgkin lymphoma (HL). Within this group, 94 patients had high-grade NHL, while 110 had classical Hodgkin lymphoma (CHL). The B-cell lymphomas, both high-grade and low-grade, accounted for 110 cases. Among the HL patients, 110 were treated with ABVD, while 90 NHL patients were candidates for R-CHOP.

**Fig. 3:**
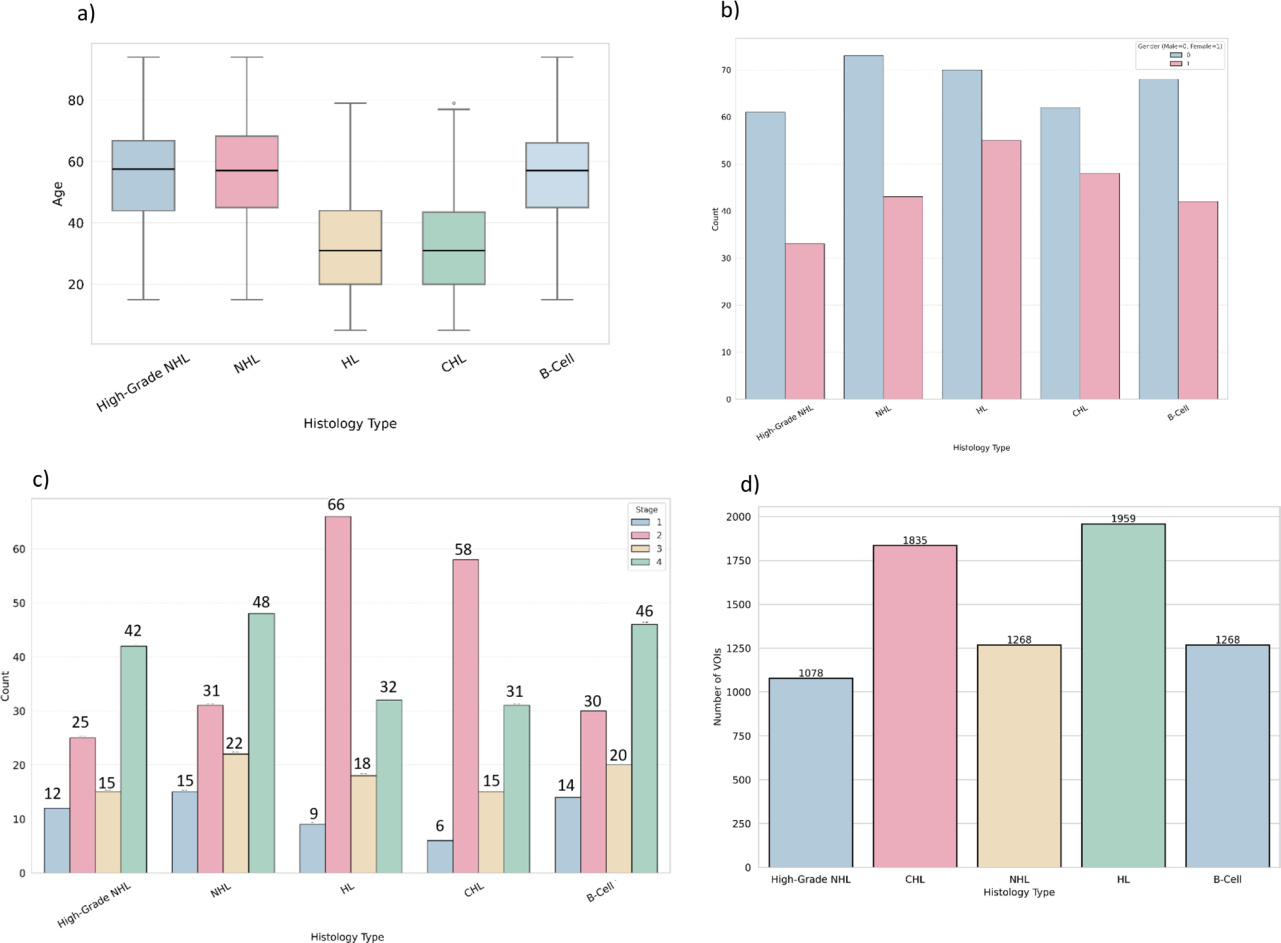
Age distribution by lymphoma type, showing older patients in high-grade NHL compared to CHL and HL. **b** Gender distribution by lymphoma type, with a higher proportion of male patients across all types. **c** Stage distribution by lymphoma type, highlighting more advanced stages in NHL and high-grade lymphomas, with a more balanced distribution in CHL. **d** Number of VOIs based on lymphoma type. * Abbreviations*: NHL: Non-Hodgkin Lymphoma; HL: Hodgkin Lymphoma; CHL: Classical Hodgkin Lymphoma; ABVD: Adriamycin, Bleomycin, Vinblastine, Dacarbazine; R-CHOP: Rituximab, Cyclophosphamide, Hydroxydaunorubicin, Oncovin (Vincristine), Prednisone

Figure 3A shows the age distribution by lymphoma type, revealing that patients with high-grade NHL tend to be older compared to those with CHL and HL overall. Figure 3B highlights the gender distribution, showing a higher proportion of male patients across all lymphoma types. Figure 3C presents the stage distribution by lymphoma type. For NHL, there is a clear distinction between early-stage and advanced-stage cases, with more patients at advanced stages. A similar pattern is seen in high-grade lymphoma, where advanced stages are more common. In contrast, classical Hodgkin lymphoma shows a more balanced distribution, with a slight majority of patients in the early stages.

Table 2 shows the age distribution across the training, internal test, and external test datasets for different types of lymphoma and treatment candidates. As shown in the table, there are significant differences between the groups. However, within each group across the three datasets (train, internal test, and external test), there are no clear, meaningful differences. In the R-CHOP group, the mean age was 50.71 years in the training set, 53.36 years in the internal test set, and 60.86 years in the external test set. By contrast, in the ABVD group, the mean age was 31.15 years in the training set, 26.44 years in the internal test set, and 36.94 years in the external test set. This suggests the importance of factors such as age in classifying and distinguishing between the groups. A similar pattern is observed across other types of lymphoma in the table, highlighting the role of age in disease onset and its influence on treatment outcomes and predictions.

**Table 2:** Age distribution in training, internal, and external test for different classifiers: ABVD vs. R-CHOP candidate, B cell vs. others, NHL vs. HL, high grade NHL vs. HL, high grade NHL vs. CHL

Classifier	Set	Type	Mean	Std	Median	25th_Percentile	75th_Percentile
ABVD Vs. R_CHOP Candidate	Train	R_CHOP	50.71429	18.49098	53	39.25	63.75
	Train	ABVD	31.14516	16.26406	29.5	20	38.5
	Internal test	R_CHOP	53.36364	16.48195	55	44	60.5
	Internal test	ABVD	26.4375	18.25365	24.5	15.25	33
	External test	R_CHOP	60.86111	16.84578	61	52.75	71
	External test	ABVD	36.93939	15.95685	35	25	51
B cell vs others	Train	others	33.79452	18.14981	31	21	45
	Train	B cell	52.77358	16.71784	55	45	62
	Internal test	others	26.44444	15.33248	21	17.25	33.75
	Internal test	B cell	48.14286	19.35839	48.5	33.75	62.25
	External test	others	36.975	15.98796	33.5	25.75	48
	External test	B cell	60.11628	16.11744	61	53	70
NHL vs HL	Train	HL	31.5942	16.3125	31	21	39
	Train	NHL	51.84211	17.27073	52	43	63
	Internal test	HL	27.88889	17.13918	21	17	33
	Internal Test	NHL	55.5	17.86595	58.5	41.75	69
	External test	HL	36.13158	15.3397	32.5	25.25	46.5
	External test	NHL	59.8	16.32706	61	52	70
High grade NHL vs.HL	Train	HL	31.5942	16.3125	31	21	39
	Train	High grade non-HL	49.90909	17.65275	52	39	62.25
	Internal test	HL	27.88889	17.13918	21	17	33
	Internal test	High grade NHL	55.36364	17.99596	55	49.5	63
	External test	HL	36.13158	15.3397	32.5	25.25	46.5
	External test	High grade NHL	59.325	17.11108	61	48.75	70
High grade NHL vs. CHL	Train	CHL	28.3871	15.90789	28	17.25	33
	Train	High grade NHL	49.90909	17.65275	52	39	62.25
	Internal test	CHL	37.125	18.27156	40	22.75	52
	Internal test	High grade NHL	55.36364	17.99596	55	49.5	63
	External test	CHL	36.93939	15.95685	35	25	51
	External test	High grade NHL	59.325	17.11108	61	48.75	70

### Radiomics-demographic combined model

**Fig. 4:**
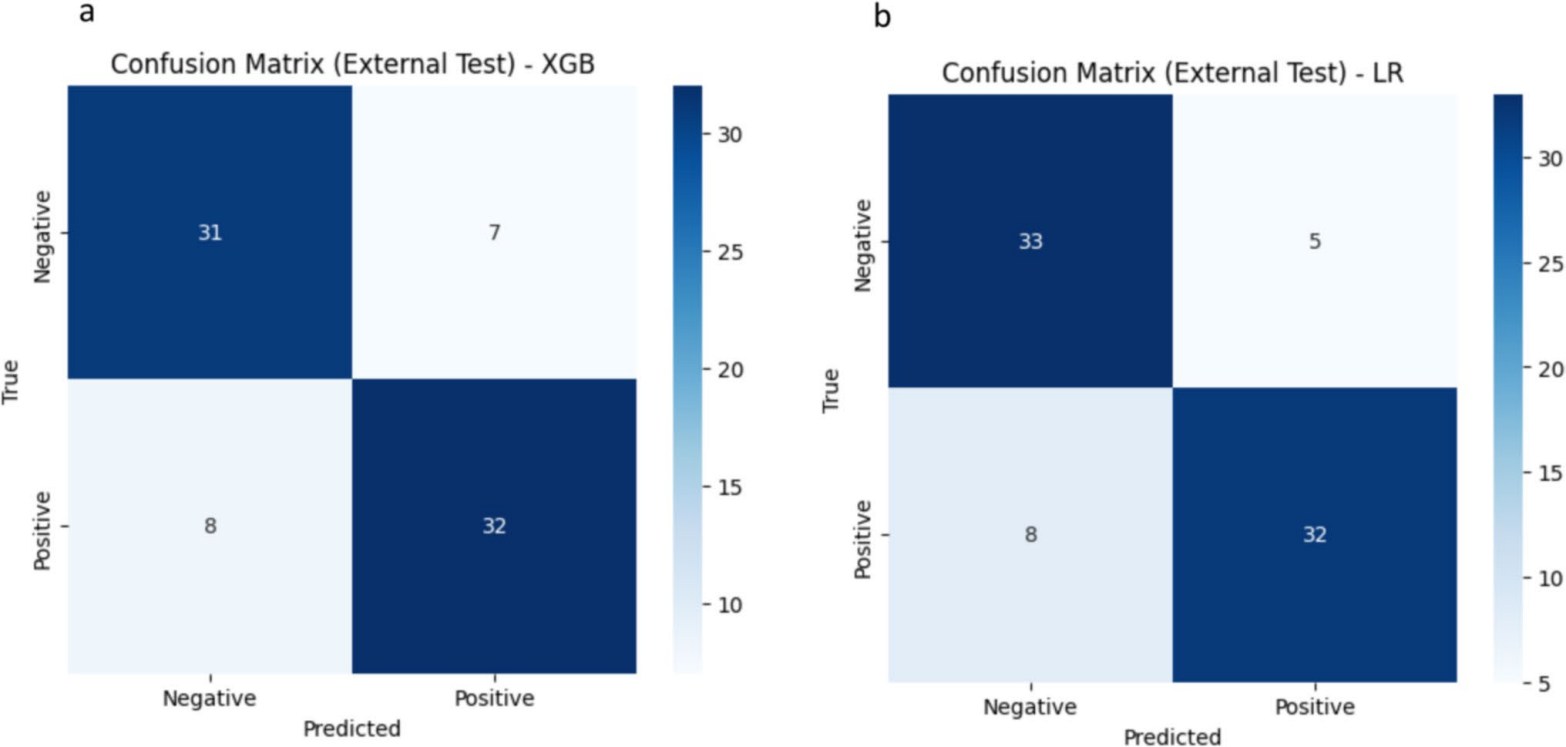
Confusion matrix for external testing of the best model in classification of high-grade NHL vs. HL using: **a** nodal + extra-nodal radiomics +  age, **b** nodal radiomics +  age

**Fig. 5:**
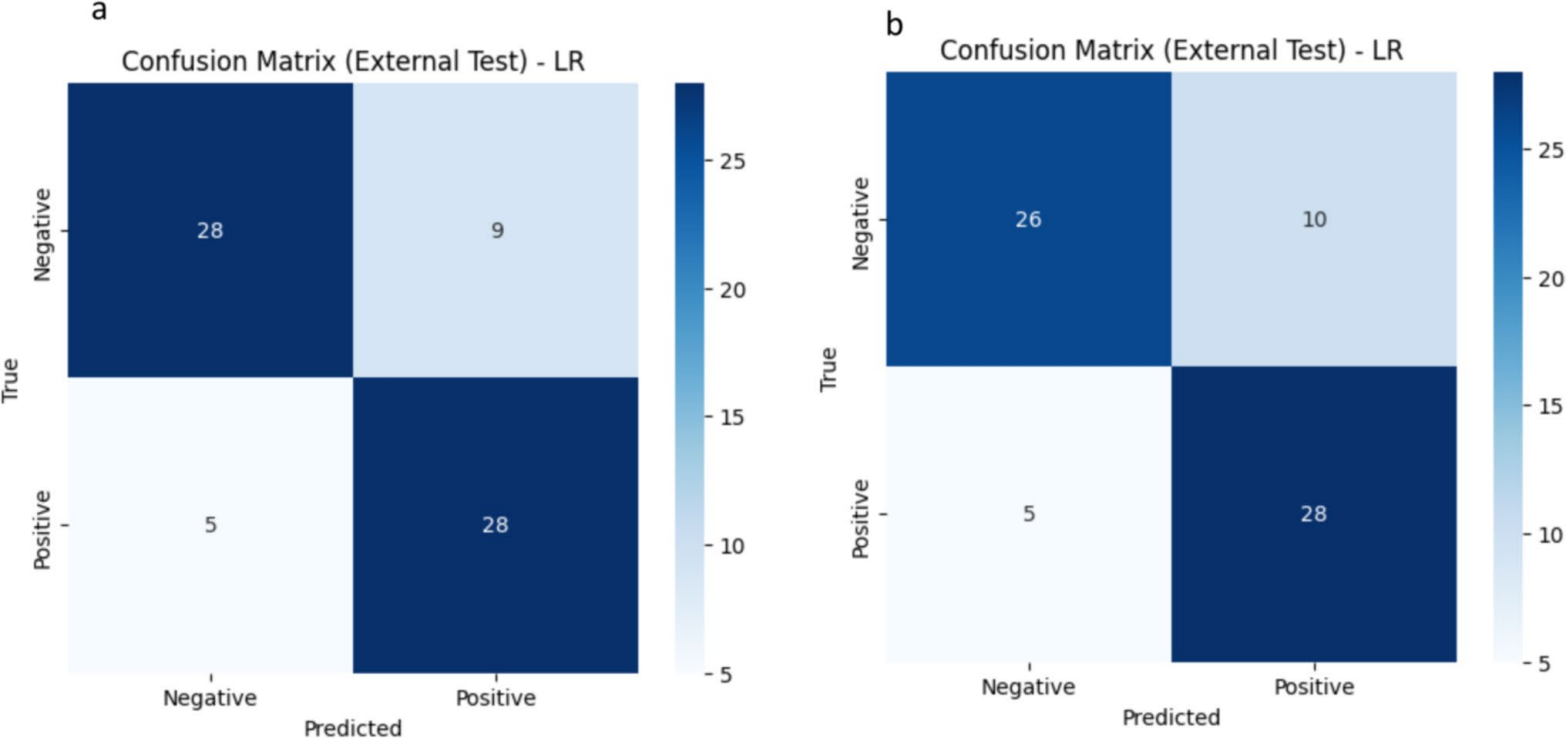
Confusion matrix for external testing of the best model in classification of ABVD Vs. R_CHOP candidate using: **a** nodal + extra-nodal radiomics + age, **b** nodal radiomics + age

**Fig. 6:**
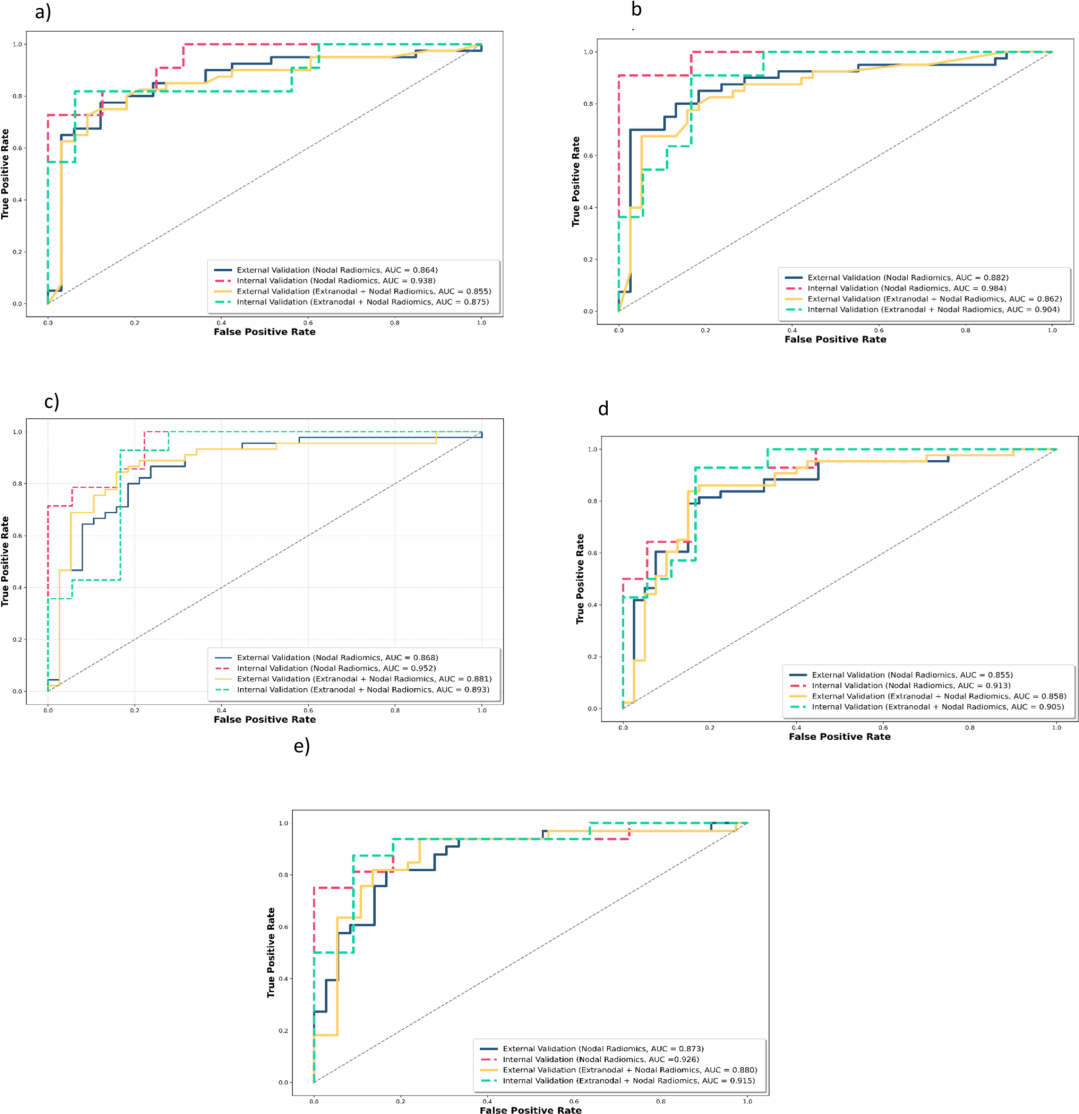
Receiver operating characteristic (ROC) curves for internal and external validation of two radiomic models: nodal and nodal plus extranodal radiomics. **A** shows High-Grade NHL vs. CHL, **B** presents High-Grade NHL vs. HL, **C** illustrates NHL vs. HL, **D** displays B-cell lymphoma vs. other lymphomas, and **E** demonstrates ABVD vs. R-CHOP candidates

A total of 107 radiomic features were extracted from 3794 segmented lesions, including 1078 high-grade NHL, 1835 CHL, 1268 NHL, 1959 HL, and 1268 B-cell lymphomas, as illustrated in Fig. 3d. These features include 14 shape features and 93 from the SUV map. Table 2 shows the performance of the best combined model, comparing the model based on nodal radiomics and the model based on both nodal and extranodal radiomics for differentiating between various lymphoma subtypes and distinguishing between the two treatment groups: ABVD candidates and R-CHOP candidates. Among demographic features, only age passed the feature selection process. The model with the highest area under the curve (AUC) was selected as the best combined model The best model for each classifier, along with the input features and classification reports, is presented in Table 4 for the external test set. Additional results for other classifiers, such as RF, XGB, and LR, are provided in the supplementary material, covering both the internal and external test sets. The cross-validation results for the training set, focusing on the best model for each classifier, are shown in Table 3. Figures 4 and 5 display the confusion matrices for the external testing of the best models in the classification of High-Grade NHL vs. HL and ABVD vs. R-CHOP candidate, respectively, using Nodal plus Extra-Nodal Radiomics and Age, as well as Nodal Radiomics plus Age. The confusion matrices for the remaining classifiers are available in the supplementary material. Furthermore, the receiver operating characteristic (ROC) curves for High-Grade NHL versus CHL and High-Grade NHL versus HL are displayed in Fig. 6A and B, respectively. The ROC curves for NHL and HL are shown in Fig. 6C, while the ROC curves for B-cell lymphoma and other lymphomas are shown in Fig. 6D. Last but not least, the ROC curves for ABVD versus R-CHOP candidates are presented in Fig. 6E.

The corresponding Demographic-Radiomic Score (Dem-Rad Score) for differentiating Hodgkin versus NHL, based on the best-performing nodal model, is provided in Eq. (1), while the formula for distinguishing ABVD and R-CHOP candidates is shown in Eq. (2). Rad Score formulas for the other classifiers are available in the Appendix.

1 $$\begin{gathered} Dem - Rad{\text{ }}Score{\text{ }}\left( {NHL{\text{ }}vs.{\text{ }}HL} \right) \hfill \\ ={\text{ }}0.91776{\text{ }} \times {\text{ }}Age{\text{ }} \hfill \\ + {\text{ }}0.22850{\text{ }} \times {\text{ }}MEAN \hfill \\ ::glszm:Gray\,Level\,Variance:TLR{\text{ }} \hfill \\ + {\text{ }}0.19985{\text{ }} \times MAX \hfill \\ ::glszm:Size\,Zone\,Non\,Uniformity:TLR{\text{ }} \hfill \\ + {\text{ }}0.17871{\text{ }} \times {\text{ }}MEAN \hfill \\ ::glcm:Contrast:TLR{\text{ }} \hfill \\ + 0.16742{\text{ }} \times {\text{ }}MAX \hfill \\ ::glrlm:Long\,Run\,High\,Gray\,Level\,Emphasis:TLR{\text{ }} \hfill \\ + {\text{ }}0.16106{\text{ }} \times MEAN \hfill \\ ::glcm:Difference\,Variance:TLR{\text{ }} \hfill \\ + {\text{ }}0.15775{\text{ }} \times {\text{ }}MEAN \hfill \\ ::glrlm:Gray\,Level\,Variance:TLR \hfill \\ + {\text{ }}0.10489{\text{ }} \times {\text{ }}MAX \hfill \\ ::glszm:High\,Gray\,Level\,Zone\,Emphasis:TLR{\text{ }} \hfill \\ + {\text{ }}0.09061{\text{ }} \times MAX \hfill \\ ::glcm:Contrast:TLR{\text{ }} \hfill \\ + {\text{ }}0.06757{\text{ }} \times {\text{ }}MAX \hfill \\ ::glcm:Difference\,Variance:TLR \hfill \\ + {\text{ }}... \hfill \\ \end{gathered} $$

2 $$\begin{gathered} Dem - Rad{\text{ }}Score{\text{ }}(ABVD{\text{ }}Vs.{\text{ }}R\_CHOP{\text{ }}Candidate){\text{ }} \hfill \\ = {\text{ }}0.60839824{\text{ }} \times {\text{ }}MEAN \hfill \\ ::first\,order:Range:TLR{\text{ }} \hfill \\ + {\text{ }}0.376862465{\text{ }} \times {\text{ }}MAX \hfill \\ ::glcm:Joint Average:TLR{\text{ }} \hfill \\ + \;0.376862465{\text{ }} \times {\text{ }}MAX \hfill \\ ::glcm:Sum\,Average:TLR{\text{ }} \hfill \\ + {\text{ }}0.338837815{\text{ }} \times MEDIAN \hfill \\ ::first\,order:Range:TLR{\text{ }} \hfill \\ + {\text{ }}0.137302474{\text{ }} \times MAX \hfill \\ ::first\,order:Mean\,Absolute\,Deviation:TLR{\text{ }} \hfill \\ + {\text{ }}0.124877362{\text{ }} \times {\text{ }} - {\text{ }}0.010622623{\text{ }} \hfill \\ \times MEDIAN::first\,order:Maximum:TLR{\text{ }} \hfill \\ - {\text{ }}0.043277459{\text{ }} \times MAX \hfill \\ ::glrlm:High\,Gray\,Level\,Run\,Emphasis:TLR{\text{ }} \hfill \\ - {\text{ }}0.07733647{\text{ }} \times MEDIAN \hfill \\ ::first\,order:Interquartile\,Range:TLR{\text{ }} \hfill \\ - {\text{ }}0.084722566{\text{ }} + {\text{ }}... \hfill \\ \end{gathered} $$

**Table 3:** Performance comparison of different lymphoma classification tasks across CV training set(Mean ± Std)

		Nodal + age	Extra nodal + nodal + age	
Task	Metric	CV Training (Mean ± Std)		CV Training (Mean ± Std)
High grade NHL vs.CHL	Accuracy	0.755 ± 0.051		0.807 ± 0.065
	AUC	0.825 ± 0.086		0.901 ± 0.106
	Sensitivity	0.614 ± 0.165		0.603 ± 0.216
	Specificity	0.854 ± 0.098		0.951 ± 0.066
High grade vs.HL	Accuracy	0.744 ± 0.118		0.771 ± 0.083
	AUC	0.731 ± 0.179		0.877 ± 0.049
	Sensitivity	0.525 ± 0.233		0.572 ± 0.172
	Specificity	0.886 ± 0.097		0.900 ± 0.086
NHL vs HL	Accuracy	0.786 ± 0.078		0.802 ± 0.049
	AUC	0.866 ± 0.061		0.875 ± 0.042
	Sensitivity	0.688 ± 0.202		0.708 ± 0.150
	Specificity	0.870 ± 0.094		0.885 ± 0.085
B cell vs others	Accuracy	0.754 ± 0.071		0.777 ± 0.056
	AUC	0.814 ± 0.063		0.822 ± 0.039
	Sensitivity	0.640 ± 0.097		0.715 ± 0.140
	Specificity	0.835 ± 0.124		0.821 ± 0.072
ABVD Vs. R_CHOP Candidate	Accuracy	0.778 ± 0.085		0.788 ± 0.120
	AUC	0.860 ± 0.101		0.852 ± 0.146
	Sensitivity	0.918 ± 0.053		0.868 ± 0.114
	Specificity	0.569 ± 0.228		0.667 ± 0.148

CHL: classical hodgkin Lymphoma; NHL: Non-Hodgkin Lymphoma; HL: hodgkin Lymphoma; ABVD: Adriamycin, Bleomycin, Vinblastine, Dacarbazine; R-CHOP: Rituximab, Cyclophosphamide, Hydroxydaunorubicin, oncovin (Vincristine), Prednisone; CV: Cross-Validation; std: standard deviation

**Table 4:** Classification report for external test across different lymphoma classification Tasks

Classifier	Sample size	Input	Algorithm	Lymphoma subtype	precision	Recall	F1-Score	Accuracy
NHL vs HL		Extra Nodal + Nodal + Age	Logistic regression	HL	0.767	0.868	0.815	0.819
				NHL	0.875	0.778	0.824	
	NHL: 116, HL: 125			Macro Average	0.821	0.823	0.819	
				Weighted Average	0.826	0.819	0.820	
				HL	0.775	0.816	0.795	0.807
		Nodal + Age	Logistic regression	NHL	0.837	0.800	0.818	
				Macro Average	0.806	0.808	0.807	
				Weighted Average	0.809	0.807	0.808	
High grade NHL vs. CHL		Extra Nodal + Nodal + Age	XGB	CHL	0.681	0.970	0.800	0.781
	High-grade NHL: 94, CHL: 110			High grade NHL	0.962	0.625	0.758	
				Macro Average	0.821	0.797	0.779	
				Weighted Average	0.835	0.781	0.777	
				CHL	0.765	0.788	0.776	0.795
		Nodal + Age	Logistic regression	High grade NHL	0.821	0.800	0.810	
				Macro Average	0.793	0.794	0.793	
				Weighted Average	0.795	0.795	0.795	
				HL	0.729	0.921	0.814	
High grade NHL vs. HL		Extra Nodal + Nodal + Age	XGB	High grade NHL	0.900	0.675	0.771	0.795
	High-grade NHL: 94, HL: 125			Macro Average	0.815	0.798	0.793	
				Weighted Average	0.817	0.795	0.792	
				HL	0.805	0.868	0.835	
		Nodal + Age	Logistic regression	High grade NHL	0.865	0.800	0.831	0.833
				Macro Average	0.835	0.834	0.833	
				Weighted Average	0.836	0.833	0.833	
				B Cell	0.85	0.791	0.819	
B cell vs. others			Logistic regression	Others	0.791	0.85	0.819	0.819
	B-cell: 110, Others: remaining patients	Extra Nodal + Nodal + Age		Macro Average	0.82	0.82	0.819	
				Weighted Average	0.821	0.819	0.819	0.819
				B Cell	0.85	0.791	0.819	
		Nodal + Age	Logistic regression	Others	0.791	0.85	0.819	
				Macro Average	0.82	0.82	0.819	
				Weighted Average	0.821	0.819	0.819	
				R_CHOP	0.848	0.757	0.800	
ABVD Vs. R_CHOP			Logistic regression	ABVD	0.757	0.848	0.80	
	ABVD: 110, R-CHOP: 90	Extra Nodal + Nodal + Age		Macro Average	0.803	0.803	0.800	0.800
				Weighted Average	0.805	0.800	0.800	
				R_CHOP	0.839	0.722	0.776	
		Nodal + Age	Logistic regression	ABVD	0.737	0.848	0.789	0.783
				Macro Average	0.788	0.785	0.782	
				Weighted Average	0.790	0.783	0.782	

CHL: classical hodgkin lymphoma; NHL: Non-Hodgkin lymphoma; HL: hodgkin lymphoma; ABVD: Adriamycin, Bleomycin, Vinblastine, Dacarbazine; R-CHOP: Rituximab, Cyclophosphamide, Hydroxydaunorubicin, oncovin (Vincristine), prednisone

## Discussion

In this study, we investigated the potential of combining baseline ^18^F-FDG PET radiomics with demographic features, using machine learning, to classify histopathological subtypes of lymphoma and distinguish between ABVD and R-CHOP candidates for first-line therapy. We employed a multi-center framework, including patients from various geographical and ethnic backgrounds, to fill the existing gaps in the literature. Furthermore, we investigated whether the inclusion of extranodal radiomics in addition to nodal radiomics could enhance the predictive performance of our model, considering the systemic nature of lymphoma.

In our study, the optimal models for each classifier, as presented in Table 4, reveal that incorporating extra-nodal radiomic features alongside nodal radiomic features and age generally improved classifier performance for certain lymphoma subtypes. Specifically, in the NHL vs. HL classification, accuracy increased from 0.807–0.819, while precision for NHL classification improved from 0.837–0.875. However, precision for HL classification decreased slightly, from 0.775–0.768.

For CHL classification, precision decreased from 0.765–0.681, whereas precision for high-grade NHL increased substantially from 0.821–0.962. This suggests that extra-nodal features were particularly beneficial for the classification of high-grade NHL, notably aiding in distinguishing it from other types, such as CHL.

In the High-grade NHL vs. HL classification, precision for High grade NHL increased from 0.865–0.900, indicating a notable improvement in identifying this group of lymphomas with the addition of extra-nodal radiomic features. This increase in precision may be due to the positive impact of these features in better distinguishing high-grade NHL from other lymphoma types. In contrast, precision for HL decreased from 0.805–0.729, suggesting reduced classifier performance in identifying this group with the addition of extra-nodal features. This decrease may indicate that extra-nodal features were not as effective in distinguishing between High grade NHL and HL, or that the added complexity of these features reduced the classifier’s ability to accurately identify HL.

In the B cell vs. others classification, the inclusion of extra-nodal features had minimal impact, with accuracy remaining at 0.819. The macro and weighted averages for precision, recall, and F1-score also remained unchanged, indicating that these additional features did not notably alter the classifier’s performance for this comparison.

In summary, incorporating extranodal radiomic features substantially improved classifier performance for high-grade NHL, reflecting the aggressive nature and widespread involvement of extranodal sites in this subtype. These features capture tumor heterogeneity and spatial distribution beyond nodal regions, providing the model with richer information to improve diagnostic accuracy. Radiomic features, such as GrayLevelVariance, SizeZoneNonUniformity, and Contrast act as imaging proxies for microstructural tumor heterogeneity. In contrast, for subtypes like classical Hodgkin lymphoma, which typically show limited extranodal spread, adding these features provides little additional information and may introduce minor noise, slightly reducing model performance. Thus, the impact of extra-nodal features is strongly dependent on the biological behavior and dissemination patterns of each lymphoma subtype rather than the mere presence of the features themselves.

When differentiating between ABVD and R-CHOP treatment regimens, however, the addition of extra-nodal radiomic features notably enhanced the classifier’s performance. The model incorporating extra-nodal features achieved an accuracy of 0.839, compared to 0.783 for the model using only nodal radiomics and age. For R-CHOP, the inclusion of extra-nodal features improved the F1-Score from 0.776–0.800, indicating better balance between precision and recall. This demonstrates that extra-nodal radiomic features enhance the classifier’s ability to identify true positives, leading to more robust performance for R-CHOP classification.

Similarly, for ABVD, the inclusion of extra-nodal features increased the F1-Score from 0.789–0.800, reflecting a slight improvement in performance. While ABVD had a relatively strong F1-Score with just nodal radiomics and age, adding extra-nodal features provided better overall balance in predicting ABVD cases. The improved accuracy and F1-Scores for both regimens underscore the value of incorporating extra-nodal radiomic data, enhancing the model’s ability to differentiate between the two treatment options.

While extra-nodal features improve classifier performance in treatment decision-making, their impact on subtype classification varies by lymphoma type. They provide greater benefits for high-grade NHL, but have less impact on Hodgkin lymphoma (HL), classic Hodgkin lymphoma (CHL), and B cell lymphoma. Since lymphoma is a systemic disease [28] that can impact multiple areas, incorporating extranodal information allows to get a more complete picture of the disease. Medical oncologists may find these features helpful because they provide insights into how the tumor behaves overall, which can aid in selecting the best treatment for patients. As this is the first study exploring this approach, it’s clear that more research is needed to confirm the findings and dig deeper into this topic.

In one study, radiomic features from ^18^F-FDG PET/CT achieved an AUC of 0.86 and 80% accuracy in distinguishing follicular lymphoma from diffuse large B-cell lymphoma [19], outperforming the SUV_max_-based model, which had an AUC of 0.79 and 70% accuracy. However, their focus on the five highest SUV lesions may have skewed their results toward higher SUV values. In our study, we tackled this issue by including lesions from every nodal station. While we can’t directly compare our results due to the different approaches taken, we believe future research should explore which method works best: relying on high SUV_max_ lesions or considering all lesions from each nodal station as a single tumor.

Additionally, Lovinfosse et al. [20] reported an AUC of 0.95 for differentiating Hodgkin lymphoma from DLBCL, which is similar to our classifier for high-grade NHL versus HL, where we achieved an AUC of 0.984 in the internal test and 0.882 in the external test. While our results are promising, we recognize that our lesion selection method may differ from others. Hence, we suggest that our approach might be more effective, although further validation is still needed. Comparing different tumor selection methods in cancers with multiple involvements, like lymphoma, could help confirm our findings. Ultimately, as the first to explore this area, we are thrilled with the achieved results. We encourage other researchers to try our approach so we can all learn more about the best ways to select tumors.

In a separate study, PET radiomics reached an accuracy of 83% for both DLBCL and follicular lymphoma, while the reported accuracies for Hodgkin lymphoma and mantle cell lymphoma were 94% and 81%, respectively [21]. Furthermore, in another investigation aimed at distinguishing gray zone lymphomas, the AUC was 0.68 [0.59, 0.66], compared to 0.77 for SUV_max_. This variation may be attributed to the smaller sample size of the gray zone group, which included only 9 patients [18].

As shown in Eq. 1 and the other equations in the appendix, age is an important demographic feature for subtype differentiation. However, in treatment decision-making, as indicated in Eq. 2, the radiomic score for ABVD candidates versus R-CHOP candidates suggests that age is not a significant factor, while radiomic features are important in this context. This may be because medical oncologists tend to select treatment plans based more on lymphoma subtype and other factors, such as the patient’s medical history—especially a history of heart disease—rather than on age alone. Since our gold standard is based on the opinions of oncologists, it reflects a preference for using clinical characteristics over age in their treatment decisions.

To further explore the clinical interpretability of our models, we derived the Dem-Rad Score for each classification task, which combines key radiomic features with demographic data. For example, the Dem-Rad Score for NHL vs. HL (Eq. 1) incorporates age and the most predictive radiomic features, such as GrayLevelVariance, SizeZoneNonUniformity, Contrast, LongRunHighGrayLevelEmphasis, and DifferenceVariance, with their corresponding coefficients indicating their relative contribution. Similarly, the Dem-Rad Score for ABVD vs. R-CHOP candidates (Eq. 2) combines first-order and texture features, including Range, JointAverage, SumAverage, MeanAbsoluteDeviation, and HighGrayLevelRunEmphasis, to capture tumor heterogeneity relevant for treatment stratification.

Among clinical variables, age was retained because it is strongly associated with lymphoma subtypes, with younger patients typically presenting with HL and older patients with NHL, reflecting established epidemiological patterns. In contrast, other clinical features, such as gender or disease stage, did not improve model performance. Gender showed no significant association with lymphoma subtype in our dataset, and disease stage, while clinically relevant for prognosis, was less informative for distinguishing subtypes when the rich radiomic features were included.

The radiomic features provide non-invasive surrogates for tumor heterogeneity and pathophysiological characteristics. For NHL vs. HL, GrayLevelVariance captures the variability of gray-level intensities, reflecting differences in cellularity or metabolic activity. SizeZoneNonUniformity measures variability in size zones, with higher values indicating more heterogeneous tissue architecture, which may correlate with tumor aggressiveness. Contrast reflects local intensity differences, capturing structural irregularities, such as necrotic or fibrotic regions. LongRunHighGrayLevelEmphasis identifies long contiguous runs of high-intensity voxels, indicating regions of high metabolic activity. DifferenceVariance quantifies variation between neighboring voxel intensities, capturing subtle heterogeneity patterns within the tumor. For ABVD vs. R-CHOP candidates, Range measures the spread of voxel intensities, reflecting heterogeneity in tumor metabolism. JointAverage and SumAverage summarize intensity relationships, highlighting patterns of local texture variation. MeanAbsoluteDeviation captures average deviation from the mean intensity, indicating intra-tumoral variability, while HighGrayLevelRunEmphasis identifies runs of high-intensity voxels, associated with hypermetabolic regions.

By integrating these features with age into the Dem-Rad Score, the model captures imaging patterns that may not be apparent on visual assessment alone, supporting the concept of a “virtual biopsy.” These features, together with supplementary Fig. 11 (panels A–D), Eqs. 1 and 2, and additional classifiers provided in the Appendix, highlight the most predictive and clinically relevant imaging biomarkers for lymphoma subtype classification and treatment stratification.

While our study benefits from a multi-center dataset including diverse lymphoma subtypes and patients from different ethnic backgrounds, some limitations remain. First, although we included two PET/CT scanners (GE Discovery and Siemens Biograph), both centers are located in Iran, and differences in imaging protocols and patient demographics may limit the generalizability of radiomic models to other populations or scanners. Second, as a retrospective study, some relevant clinical factors influencing treatment decisions and outcomes may not have been captured. Third, variability in pathological assessment of lymphoma subtypes could contribute to inconsistencies in the dataset. These factors should be considered when interpreting the results and their potential clinical application.

## Conclusion

In conclusion, this study highlights the potential of using ^18^F-FDG PET radiomics alongside demographic features to classify lymphoma subtypes and help guide treatment decisions. Although extra-nodal features had a notable impact on certain subtypes, such as high-grade NHL, their effect was minimal or negligible for other subtypes. However, they notably improved treatment classification in distinguishing between ABVD and R-CHOP candidates, particularly for R-CHOP candidates, underscoring their importance in treatment selection. This non-invasive approach could eventually reduce the need for repeated biopsies, especially during disease transformations, and contribute to more personalized treatments. Moreover, the model’s ability to differentiate ABVD from R-CHOP candidates offers a helpful tool for making informed decisions about treatment. While our findings are promising, further validation is necessary, given the challenges of varying imaging protocols across centers and differing opinions among pathologists. Future research should focus on refining tumor selection methods and confirming these results in larger, more diverse patient groups.

## Supplementary Information

Below is the link to the electronic supplementary material.

Supplementary Material 1.

## Data Availability

The corresponding author may provide the data and materials used in this study upon reasonable request.

## Appendix

$$ \begin{gathered} Dem - Rad{\text{ }}Score\left( {for{\text{ }}High - Grade{\text{ }}NHL{\text{ }}vs.CHL} \right) \hfill \\ = {\text{ }}10.67390479{\text{ }} \times {\text{ }}MEAN \hfill \\ ::glszm:Gray\,Level\,Variance:TLR{\text{ }} \hfill \\ + {\text{ }}6.782708797{\text{ }} \times {\text{ }}MEAN \hfill \\ ::glcm:Difference\,Variance:TLR{\text{ }} \hfill \\ + \;6.334659382{\text{ }} \times {\text{ }}MEDIAN \hfill \\ ::first\,order:Mean\,Absolute\,Deviation:TLR{\text{ }} \hfill \\ + {\text{ }}2.560540613{\text{ }} \times \;MEAN \hfill \\ ::glcm:Contrast:TLR{\text{ }} \hfill \\ + {\text{ }}2.270324057{\text{ }} \times {\text{ }}MIN \hfill \\ ::glrlm:Run\,Entropy:TLR{\text{ }} \hfill \\ + {\text{ }}1.823281517{\text{ }} \times MAX \hfill \\ ::glszm:High\,Gray\,Level\,Zone\,Emphasis:TLR{\text{ }} \hfill \\ + {\text{ }}1.595680939{\text{ }} \times {\text{ }}Age{\text{ }} \hfill \\ + {\text{ }}0.057479781{\text{ }} \times MEAN \hfill \\ ::first\,order:90Percentile:TLR{\text{ }} \hfill \\ - {\text{ }}0.184575291{\text{ }} \times {\text{ }}MEDIAN \hfill \\ ::first\,order:Range:TLR{\text{ }} \hfill \\ - \;0.294395162{\text{ }} \times {\text{ }}MEAN \hfill \\ ::gldm:Small\,Dependence\,Emphasis:TLR \hfill \\ + {\text{ }}... \hfill \\ \end{gathered} $$

$$\begin{gathered} Dem - Rad{\text{ }}Score\left( {for{\text{ }}High - Grade{\text{ }}NHL{\text{ }}vs. HL} \right) \hfill \\ = {\text{ }}6.635283008{\text{ }} \times MEAN \hfill \\ ::glrlm:ShortRunHighGrayLevelEmphasis \hfill \\ :TLR{\text{ }} + {\text{ }}5.537143115{\text{ }} \hfill \\ \times MEDIAN::first\,order:Robust\,Mean\,Absolute\,Deviation \hfill \\ :TLR{\text{ }} + {\text{ }}3.523025559{\text{ }} \hfill \\ \times MEAN::glrlm:Run\,Entropy:TLR{\text{ }} \hfill \\ + {\text{ }}1.823274139{\text{ }} \times {\text{ }}MEDIAN \hfill \\ ::first\,order:Maximum:TLR{\text{ }} \hfill \\ + \;1.743252694{\text{ }} \times {\text{ }}Age{\text{ }} \hfill \\ + {\text{ }}0.409273738{\text{ }} \times {\text{ }}MEAN \hfill \\ ::glcm:Difference\,Variance:TLR{\text{ }} \hfill \\ + {\text{ }}0.219053137{\text{ }} \times MAX \hfill \\ ::glrlm:Gray\,Level\,Variance:TLR{\text{ }} \hfill \\ + {\text{ }}0.167566703{\text{ }} \times MEDIAN \hfill \\ ::first\,order:Mean\,Absolute\,Deviation:TLR{\text{ }} \hfill \\ + {\text{ }}0.039428023{\text{ }} \times {\text{ }}MEAN \hfill \\ ::glcm:Contrast:TLR{\text{ }} \hfill \\ - \;1.650686823{\text{ }} \times {\text{ }}MEAN \hfill \\ ::glcm:Sum\,Average:TLR{\text{ }} \hfill \\ - {\text{ }}1.807575984{\text{ }} \times {\text{ }}MEAN \hfill \\ ::glcm:Joint Average:TLR + \;... \hfill \\ \end{gathered} $$

$$ \begin{gathered} Dem - Rad{\text{ }}Score\left( {for{\text{ }}B - Cell{\text{ }}vs.{\text{ }}Others} \right) \hfill \\ = {\text{ }}1.180707871{\text{ }} \times {\text{ }}Age{\text{ }} \hfill \\ + {\text{ }}0.968453774{\text{ }} \hfill \\ \times {\text{ }}MAX::gldm \hfill \\ :Large\,Dependence\,High\,Gray\,Level\,Emphasis \hfill \\ :TLR{\text{ }} + {\text{ }}0.42293689{\text{ }} \hfill \\ \times MEAN::glrlm \hfill \\ :Short\,Run\,High\,Gray\,Level\,Emphasis \hfill \\ :TLR{\text{ }} + {\text{ }}0.418102775{\text{ }} \times MAX \hfill \\ ::gldm:Small\,Dependence\,High\,Gray\,Level\,Emphasis \hfill \\ :TLR{\text{ }} + {\text{ }}0.312861666{\text{ }} \times MEAN \hfill \\ ::first\,order:Variance:TLR{\text{ }} \hfill \\ + {\text{ }}0.264658982{\text{ }} \times {\text{ }}MAX \hfill \\ ::glszm:Gray\,Level\,Variance:TLR{\text{ }} \hfill \\ + 0.193638637{\text{ }} \times {\text{ }}MAX::first\,order \hfill \\ :Variance:TLR{\text{ }} + {\text{ }}0.158215551{\text{ }} \hfill \\ \times {\text{ }}MAX::glcm:Contrast:TLR{\text{ }} \hfill \\ + 0.147312431{\text{ }} \times {\text{ }}MAX \hfill \\ ::glrlm:Gray\,Level\,Variance:TLR{\text{ }} \hfill \\ + {\text{ }}0.127281358{\text{ }} \times MAX::glrlm \hfill \\ :Long\,Run\,High\,Gray\,Level\,Emphasis:TLR{\text{ }} \hfill \\ + {\text{ }}0.111898843{\text{ }} \times {\text{ }}MEAN::glcm \hfill \\ :Contrast:TLR{\text{ }} + 0.070591403{\text{ }} \hfill \\ \times {\text{ }}MAX::glszm:Size\,Zone\,Non\,Uniformity \hfill \\ :TLR{\text{ }} + {\text{ }}0.058857585{\text{ }} \times MAX::glrlm \hfill \\ :Short\,Run\,High\,Gray\,Level\,Emphasis \hfill \\ :TLR{\text{ }} - {\text{ }}0.076065242{\text{ }} \times MAX::gldm \hfill \\ :Gray\,Level\,Variance:TLR{\text{ }} + {\text{ }}... \hfill \\ \end{gathered} $$
