# Supplementary material for: ¹⁸F-FDG PET radiomics and machine learning for virtual biopsy and treatment decisions in lymphoma: a multicenter study
Source: Phys Eng Sci Med. 2025 Nov 20;49(1):381–95. doi: 10.1007/s13246-025-01675-2 (PMC12987794; doi:10.1007/s13246-025-01675-2)
Supplement: Supplementary file 1 — Supplementary Material 1. [file 13246_2025_1675_MOESM1_ESM.pdf]

Supplementary material

Extranodal + Nodal plus age:

1. External test:

Table 1. Classification report for (Extranodal + Nodal) radiomics combined with age in external test for differentiating NHL vs. HL.

Classifier	Class	precision	recall	f1-score
**LR (NHL vs HL)**	**HL**	0.767442	0.868421	0.814815
	**NHL**	0.875	0.777778	0.823529
	**accuracy**	0.819277	0.819277	0.819277
	**macro avg**	0.821221	0.823099	0.819172
	**weighted avg**	0.825757	0.819277	0.81954
**RF (NHL vs HL)**	**HL**	0.818182	0.710526	0.760563
	**NHL**	0.78	0.866667	0.821053
	**accuracy**	0.795181	0.795181	0.795181
	**macro avg**	0.799091	0.788596	0.790808
	**weighted avg**	0.797481	0.795181	0.793359
**XGB (NHL vs HL)**	**HL**	0.710526	0.710526	0.710526
	**NHL**	0.755556	0.755556	0.755556
	**accuracy**	0.73494	0.73494	0.73494
	**macro avg**	0.733041	0.733041	0.733041
	**weighted avg**	0.73494	0.73494	0.73494

Table 2. Classification report for (Extranodal + Nodal) radiomics combined with age in external test for differentiating High grade non-HL vs. CHL.

Classifier	Class	precision	recall	f1-score
**LR (High grade non-HL vs.CHL)**	**CHL**	0.675	0.818182	0.739726
	**High grade non-HL**	0.818182	0.675	0.739726
	**accuracy**	0.739726	0.739726	0.739726
	**macro avg**	0.746591	0.746591	0.739726
	**weighted avg**	0.753456	0.739726	0.739726
**RF (High grade non-HL vs.CHL)**	**CHL**	0.676471	0.69697	0.686567
	**High grade non-HL**	0.74359	0.725	0.734177
	**accuracy**	0.712329	0.712329	0.712329
	**macro avg**	0.71003	0.710985	0.710372
	**weighted avg**	0.713248	0.712329	0.712655
**XGB (High grade non-HL vs.CHL)**	**CHL**	0.680851	0.969697	0.8
	**High grade non-HL**	0.961538	0.625	0.757576
	**accuracy**	0.780822	0.780822	0.780822
	**macro avg**	0.821195	0.797348	0.778788
	**weighted avg**	0.834652	0.780822	0.776754

Table 3. Classification report for (Extranodal + Nodal) radiomics combined with age in external test for differentiating High grade non-HL vs. HL.

Classifier	Class	precision	recall	f1-score
**LR (High grade NHL vs.HL)**	**HL**	0.775	0.815789	0.794872
	**High grade NHL**	0.815789	0.775	0.794872
	**accuracy**	0.794872	0.794872	0.794872
	**macro avg**	0.795395	0.795395	0.794872
	**weighted avg**	0.795918	0.794872	0.794872
**RF (High grade NHL vs.HL)**	**HL**	0.75	0.789474	0.769231
	**High grade NHL**	0.789474	0.75	0.769231
	**accuracy**	0.769231	0.769231	0.769231
	**macro avg**	0.769737	0.769737	0.769231
	**weighted avg**	0.770243	0.769231	0.769231
**XGB (High grade NHL vs.HL)**	**HL**	0.729167	0.921053	0.813953
	**High grade NHL**	0.9	0.675	0.771429
	**accuracy**	0.794872	0.794872	0.794872
	**macro avg**	0.814583	0.798026	0.792691
	**weighted avg**	0.816774	0.794872	0.792146

Table 4. Classification report for (Extranodal + Nodal) radiomics combined with age in external test for differentiating B cell vs. others**.**

Classifier	Class	precision	recall	f1-score
**LR (B cell vs. others)**	**others**	0.790698	0.85	0.819277
	**B cell**	0.85	0.790698	0.819277
	**accuracy**	0.819277	0.819277	0.819277
	**macro avg**	0.820349	0.820349	0.819277
	**weighted avg**	0.821421	0.819277	0.819277
**RF (B cell vs. others)**	**others**	0.757576	0.625	0.684932
	**B cell**	0.7	0.813953	0.752688
	**accuracy**	0.722892	0.722892	0.722892
	**macro avg**	0.728788	0.719477	0.71881
	**weighted avg**	0.727747	0.722892	0.720034
**XGB (B cell vs. others)**	**others**	0.666667	0.85	0.747253
	**B cell**	0.8125	0.604651	0.693333
	**accuracy**	0.722892	0.722892	0.722892
	**macro avg**	0.739583	0.727326	0.720293
	**weighted avg**	0.742219	0.722892	0.719319

Table 5. Classification report for (Extranodal + Nodal) radiomics combined with age in external test for differentiating AVBD Vs. R_CHOP Candidate.

Classifier	class	precision	recall	f1-score
**LR (AVBD Vs. R_CHOP Candidate)**	**R_CHOP**	0.848485	0.756757	0.8
	**AVBD**	0.756757	0.848485	0.8
	**accuracy**	0.8	0.8	0.8
	**macro avg**	0.802621	0.802621	0.8
	**weighted avg**	0.805242	0.8	0.8
**RF (AVBD Vs. R_CHOP Candidate)**	**R_CHOP**	0.8	0.756757	0.777778
	**AVBD**	0.742857	0.787879	0.764706
	**accuracy**	0.771429	0.771429	0.771429
	**macro avg**	0.771429	0.772318	0.771242
	**weighted avg**	0.773061	0.771429	0.771615
**XGB (AVBD Vs. R_CHOP Candidate)**	**R_CHOP**	0.875	0.756757	0.811594
	**AVBD**	0.763158	0.878788	0.816901
	**accuracy**	0.814286	0.814286	0.814286
	**macro avg**	0.819079	0.817772	0.814248
	**weighted avg**	0.822274	0.814286	0.814096

2. Internal test:

Table 6. Classification report for (Extranodal + Nodal) radiomics combined with age in internal test for differentiating NHL vs HL.

Classifier	Class	precision	recall	f1-score
**LR (NHL vs HL)**	**HL**	0.714286	0.833333	0.769231
	**NHL**	0.727273	0.571429	0.64
	**accuracy**	0.71875	0.71875	0.71875
	**macro avg**	0.720779	0.702381	0.704615
	**weighted avg**	0.719968	0.71875	0.712692
**RF (NHL vs HL)**	**HL**	0.75	0.833333	0.789474
	**NHL**	0.75	0.642857	0.692308
	**accuracy**	0.75	0.75	0.75
	**macro avg**	0.75	0.738095	0.740891
	**weighted avg**	0.75	0.75	0.746964
**XGB (NHL vs HL)**	**HL**	0.833333	0.833333	0.833333
	**NHL**	0.785714	0.785714	0.785714
	**accuracy**	0.8125	0.8125	0.8125
	**macro avg**	0.809524	0.809524	0.809524
	**weighted avg**	0.8125	0.8125	0.8125

Table 7. Classification report for (Extranodal + Nodal) radiomics combined with age in internal test for differentiating High grade non-HL vs.CHL.

Classifier	Class	precision	recall	f1-score
**LR (High grade non-HL vs.CHL)**	**CHL**	0.705882	0.75	0.727273
	**High grade non-HL**	0.6	0.545455	0.571429
	**accuracy**	0.666667	0.666667	0.666667
	**macro avg**	0.652941	0.647727	0.649351
	**weighted avg**	0.662745	0.666667	0.663781
**RF (High grade non-HL vs.CHL)**	**CHL**	0.777778	0.875	0.823529
	**High grade non-HL**	0.777778	0.636364	0.7
	**accuracy**	0.777778	0.777778	0.777778
	**macro avg**	0.777778	0.755682	0.761765
	**weighted avg**	0.777778	0.777778	0.773203
**XGB (High grade non-HL vs.CHL)**	**CHL**	0.789474	0.9375	0.857143
	**High grade non-HL**	0.875	0.636364	0.736842
	**accuracy**	0.814815	0.814815	0.814815
	**macro avg**	0.832237	0.786932	0.796992
	**weighted avg**	0.824318	0.814815	0.808131

Table 8. Classification report for (Extranodal + Nodal) radiomics combined with age in internal test for differentiating High grade non-HL vs. HL.

Classifier	Class	precision	recall	f1-score
**LR (High grade vs.HL)**	**HL**	0.8	0.888889	0.842105
	**High grade NHL**	0.777778	0.636364	0.7
	**accuracy**	0.793103	0.793103	0.793103
	**macro avg**	0.788889	0.762626	0.771053
	**weighted avg**	0.791571	0.793103	0.788203
**RF (High grade vs.HL)**	**HL**	0.727273	0.888889	0.8
	**High grade NHL**	0.714286	0.454545	0.555556
	**accuracy**	0.724138	0.724138	0.724138
	**macro avg**	0.720779	0.671717	0.677778
	**weighted avg**	0.722347	0.724138	0.70728
**XGB (High grade vs.HL)**	**HL**	0.772727	0.944444	0.85
	**High grade NHL**	0.857143	0.545455	0.666667
	**accuracy**	0.793103	0.793103	0.793103
	**macro avg**	0.814935	0.744949	0.758333
	**weighted avg**	0.804747	0.793103	0.78046

Table 9. Classification report for (Extranodal + Nodal) radiomics combined with age in internal test for differentiating B cell vs. others.

classifier	Class	precision	recall	f1-score
**LR (B cell vs. others)**	**others**	0.695652	0.888889	0.780488
	**B cell**	0.777778	0.5	0.608696
	**accuracy**	0.71875	0.71875	0.71875
	**macro avg**	0.736715	0.694444	0.694592
	**weighted avg**	0.731582	0.71875	0.705329
**RF (B cell vs. others)**	**others**	0.8	0.888889	0.842105
	**B cell**	0.833333	0.714286	0.769231
	**accuracy**	0.8125	0.8125	0.8125
	**macro avg**	0.816667	0.801587	0.805668
	**weighted avg**	0.814583	0.8125	0.810223
**XGB (B cell vs. others)**	**others**	0.727273	0.888889	0.8
	**B cell**	0.8	0.571429	0.666667
	**accuracy**	0.75	0.75	0.75
	**macro avg**	0.763636	0.730159	0.733333
	**weighted avg**	0.759091	0.75	0.741667

Table 10. Classification report for (Extranodal + Nodal) radiomics combined with age in internal test for differentiating AVBD Vs. R_CHOP Candidate.

Classifier	Class	precision	recall	f1-score
**LR (AVBD Vs. R_CHOP Candidate)**	**R_CHOP**	0.857143	0.545455	0.666667
	**AVBD**	0.75	0.9375	0.833333
	**accuracy**	0.777778	0.777778	0.777778
	**macro avg**	0.803571	0.741477	0.75
	**weighted avg**	0.793651	0.777778	0.765432
**RF (AVBD Vs. R_CHOP Candidate)**	**R_CHOP**	0.875	0.636364	0.736842
	**AVBD**	0.789474	0.9375	0.857143
	**accuracy**	0.814815	0.814815	0.814815
	**macro avg**	0.832237	0.786932	0.796992
	**weighted avg**	0.824318	0.814815	0.808131
**XGB (AVBD Vs. R_CHOP Candidate)**	**R_CHOP**	0.875	0.636364	0.736842
	**AVBD**	0.789474	0.9375	0.857143
	**accuracy**	0.814815	0.814815	0.814815
	**macro avg**	0.832237	0.786932	0.796992
	**weighted avg**	0.824318	0.814815	0.808131

2. Nodal plus age:

1.External Test

Table 11. Classification report for Nodal radiomics combined with age in external test for differentiating NHL vs HL.

Classifier	Class	precision	recall	f1-score
**LR (NHL vs HL)**	**HL**	0.775	0.815789	0.794872
	**NHL**	0.837209	0.8	0.818182
	**accuracy**	0.807229	0.807229	0.807229
	**macro avg**	0.806105	0.807895	0.806527
	**weighted avg**	0.808728	0.807229	0.80751
**RF (NHL vs HL)**	**HL**	0.658537	0.710526	0.683544
	**NHL**	0.738095	0.688889	0.712644
	**accuracy**	0.698795	0.698795	0.698795
	**macro avg**	0.698316	0.699708	0.698094
	**weighted avg**	0.701671	0.698795	0.699321
**XGB (NHL vs HL)**	**HL**	0.7	0.736842	0.717949
	**NHL**	0.767442	0.733333	0.75
	**accuracy**	0.73494	0.73494	0.73494
	**macro avg**	0.733721	0.735088	0.733974
	**weighted avg**	0.736565	0.73494	0.735326

Table 1. Classification report for Nodal radiomics combined with age in external test for differentiating High grade non-HL vs.CHL.

Classifier	Class	**precision**	**recall**	**f1-score**
**LR (High grade non-HL vs.CHL)**	**CHL**	0.764706	0.787879	0.776119
	**High grade non-HL**	0.820513	0.8	0.810127
	**accuracy**	0.794521	0.794521	0.794521
	**macro avg**	0.792609	0.793939	0.793123
	**weighted avg**	0.795285	0.794521	0.794753
**RF (High grade non-HL vs.CHL)**	**CHL**	0.724138	0.636364	0.677419
	**High grade non-HL**	0.727273	0.8	0.761905
	**accuracy**	0.726027	0.726027	0.726027
	**macro avg**	0.725705	0.718182	0.719662
	**weighted avg**	0.725856	0.726027	0.723713
**XGB (High grade non-HL vs.CHL)**	**CHL**	0.674419	0.878788	0.763158
	**High grade non-HL**	0.866667	0.65	0.742857
	**accuracy**	0.753425	0.753425	0.753425
	**macro avg**	0.770543	0.764394	0.753008
	**weighted avg**	0.77976	0.753425	0.752034

Table 2. Classification report for Nodal radiomics combined with age in external test for differentiating High grade NHL vs.HL.

**Classifier**	**Class**	**precision**	**recall**	**f1-score**
**LR (High grade NHL vs.HL)**	**HL**	0.804878	0.868421	0.835443
	**High grade NHL**	0.864865	0.8	0.831169
	**accuracy**	0.833333	0.833333	0.833333
	**macro avg**	0.834871	0.834211	0.833306
	**weighted avg**	0.835641	0.833333	0.833251
**RF (High grade NHL vs.HL)**	**HL**	0.692308	0.947368	0.8
	**High grade NHL**	0.923077	0.6	0.727273
	**accuracy**	0.769231	0.769231	0.769231
	**macro avg**	0.807692	0.773684	0.763636
	**weighted avg**	0.810651	0.769231	0.762704
**XGB (High grade NHL vs.HL)**	**HL**	0.553846	0.947368	0.699029
	**High grade NHL**	0.846154	0.275	0.415094
	**accuracy**	0.602564	0.602564	0.602564
	**macro avg**	0.7	0.611184	0.557062
	**weighted avg**	0.703748	0.602564	0.553422

Table 14. Classification report for Nodal radiomics combined with age in external test for differentiating B cell vs others.

Classifier	Class	precision	recall	f1-score
**LR (B cell vs others)**	**others**	0.790698	0.85	0.819277
	**B cell**	0.85	0.790698	0.819277
	**accuracy**	0.819277	0.819277	0.819277
	**macro avg**	0.820349	0.820349	0.819277
	**weighted avg**	0.821421	0.819277	0.819277
**RF (B cell vs others)**	**others**	0.794872	0.775	0.78481
	**B cell**	0.795455	0.813953	0.804598
	**accuracy**	0.795181	0.795181	0.795181
	**macro avg**	0.795163	0.794477	0.794704
	**weighted avg**	0.795174	0.795181	0.795062
**XGB (B cell vs others)**	**others**	0.723404	0.85	0.781609
	**B cell**	0.833333	0.697674	0.759494
	**accuracy**	0.771084	0.771084	0.771084
	**macro avg**	0.778369	0.773837	0.770551
	**weighted avg**	0.780355	0.771084	0.770152

Table 15. Classification report for Nodal radiomics combined with age in external test for differentiating AVBD Vs. R_CHOP Candidate others.

Classifier	Class	precision	recall	f1-score
**LR (AVBD Vs. R_CHOP Candidate)**	**R_CHOP**	0.83871	0.722222	0.776119
	**AVBD**	0.736842	0.848485	0.788732
	**accuracy**	0.782609	0.782609	0.782609
	**macro avg**	0.787776	0.785354	0.782426
	**weighted avg**	0.78999	0.782609	0.782152
**RF (AVBD Vs. R_CHOP Candidate)**	**R_CHOP**	0.717949	0.777778	0.746667
	**AVBD**	0.733333	0.666667	0.698413
	**accuracy**	0.724638	0.724638	0.724638
	**macro avg**	0.725641	0.722222	0.72254
	**weighted avg**	0.725307	0.724638	0.723589
**XGB (AVBD Vs. R_CHOP Candidate)**	**R_CHOP**	0.851852	0.638889	0.730159
	**AVBD**	0.690476	0.878788	0.773333
	**accuracy**	0.753623	0.753623	0.753623
	**macro avg**	0.771164	0.758838	0.751746
	**weighted avg**	0.774672	0.753623	0.750807

2.internal validation:

Table 16. Classification report for Nodal radiomics combined with age in internal test for differentiating NHL vs HL.

Classifier	Class	precision	recall	f1-score
**LR (NHL vs HL)**	**HL**	0.842105	0.888889	0.864865
	**NHL**	0.846154	0.785714	0.814815
	**accuracy**	0.84375	0.84375	0.84375
	**macro avg**	0.84413	0.837302	0.83984
	**weighted avg**	0.843877	0.84375	0.842968
**RF (NHL vs HL)**	**HL**	0.8	0.888889	0.842105
	**NHL**	0.833333	0.714286	0.769231
	**accuracy**	0.8125	0.8125	0.8125
	**macro avg**	0.816667	0.801587	0.805668
	**weighted avg**	0.814583	0.8125	0.810223
**XGB (NHL vs HL)**	**HL**	0.8	0.888889	0.842105
	**NHL**	0.833333	0.714286	0.769231
	**accuracy**	0.8125	0.8125	0.8125
	**macro avg**	0.816667	0.801587	0.805668
	**weighted avg**	0.814583	0.8125	0.810223

Table 3 Classification report for Nodal radiomics combined with age in internal test for differentiating High grade NHL vs.CHL.

Classifier	Class	precision	recall	f1-score
**LR (High grade NHL vs.CHL)**	**CHL**	0.857143	0.75	0.8
	**High grade NHL**	0.692308	0.818182	0.75
	**accuracy**	0.777778	0.777778	0.777778
	**macro avg**	0.774725	0.784091	0.775
	**weighted avg**	0.789988	0.777778	0.77963
**LR (High grade non-HL vs.CHL)**	**CHL**	0.785714	0.6875	0.733333
	**High grade NHL**	0.615385	0.727273	0.666667
	**accuracy**	0.703704	0.703704	0.703704
	**macro avg**	0.700549	0.707386	0.7
	**weighted avg**	0.716321	0.703704	0.706173
**LR (High grade non-HL vs.CHL)**	**CHL**	1	0.875	0.933333
	**High grade NHL**	0.846154	1	0.916667
	**accuracy**	0.925926	0.925926	0.925926
	**macro avg**	0.923077	0.9375	0.925
	**weighted avg**	0.937322	0.925926	0.926543

Table 18. Classification report for Nodal radiomics combined with age in internal test for differentiating High grade NHL vs. HL.

Classifier	Class	precision	recall	f1-score
LR (High grade NHL vs.HL)	HL	0.9375	0.833333	0.882353
	High grade NHL	0.769231	0.909091	0.833333
	accuracy	0.862069	0.862069	0.862069
	macro avg	0.853365	0.871212	0.857843
	weighted avg	0.873674	0.862069	0.863759
RF (High grade NHL vs.HL)	HL	0.782609	1	0.878049
	High grade NHL	1	0.545455	0.705882
	accuracy	0.827586	0.827586	0.827586
	macro avg	0.891304	0.772727	0.791966
	weighted avg	0.865067	0.827586	0.812744
XGB (High grade NHL vs.HL)	HL	0.708333	0.944444	0.809524
	High grade NHL	0.8	0.363636	0.5
	accuracy	0.724138	0.724138	0.724138
	macro avg	0.754167	0.65404	0.654762
	weighted avg	0.743103	0.724138	0.692118

Table 19.4 Classification report for Nodal radiomics combined with age in internal test for differentiating B cell vs others.

Classifier	Class	precision	recall	f1-score
LR (B cell vs others)	others	0.772727	0.944444	0.85
	B cell	0.9	0.642857	0.75
	accuracy	0.8125	0.8125	0.8125
	macro avg	0.836364	0.793651	0.8
	weighted avg	0.828409	0.8125	0.80625
RF (B cell vs others)	others	0.842105	0.888889	0.864865
	B cell	0.846154	0.785714	0.814815
	accuracy	0.84375	0.84375	0.84375
	macro avg	0.84413	0.837302	0.83984
	weighted avg	0.843877	0.84375	0.842968
XGB (B cell vs others)	others	0.761905	0.888889	0.820513
	B cell	0.818182	0.642857	0.72
	accuracy	0.78125	0.78125	0.78125
	macro avg	0.790043	0.765873	0.770256
	weighted avg	0.786526	0.78125	0.776538

Table 20. Classification report for Nodal radiomics combined with age in internal test for differentiating AVBD Vs. R_CHOP Candidate

Classifier	Class	precision	recall	f1-score
**LR (AVBD Vs. R_CHOP Candidate)**	**R_CHOP**	0.875	0.636364	0.736842
	**AVBD**	0.789474	0.9375	0.857143
	**accuracy**	0.814815	0.814815	0.814815
	**macro avg**	0.832237	0.786932	0.796992
	**weighted avg**	0.824318	0.814815	0.808131
**RF (AVBD Vs. R_CHOP Candidate)**	**R_CHOP**	0.888889	0.727273	0.8
	**AVBD**	0.833333	0.9375	0.882353
	**accuracy**	0.851852	0.851852	0.851852
	**macro avg**	0.861111	0.832386	0.841176
	**weighted avg**	0.855967	0.851852	0.848802
**XGB (AVBD Vs. R_CHOP Candidate)**	**R_CHOP**	1	0.636364	0.777778
	**AVBD**	0.8	1	0.888889
	**accuracy**	0.851852	0.851852	0.851852
	**macro avg**	0.9	0.818182	0.833333
	**weighted avg**	0.881481	0.851852	0.843621

Confusion matrix:

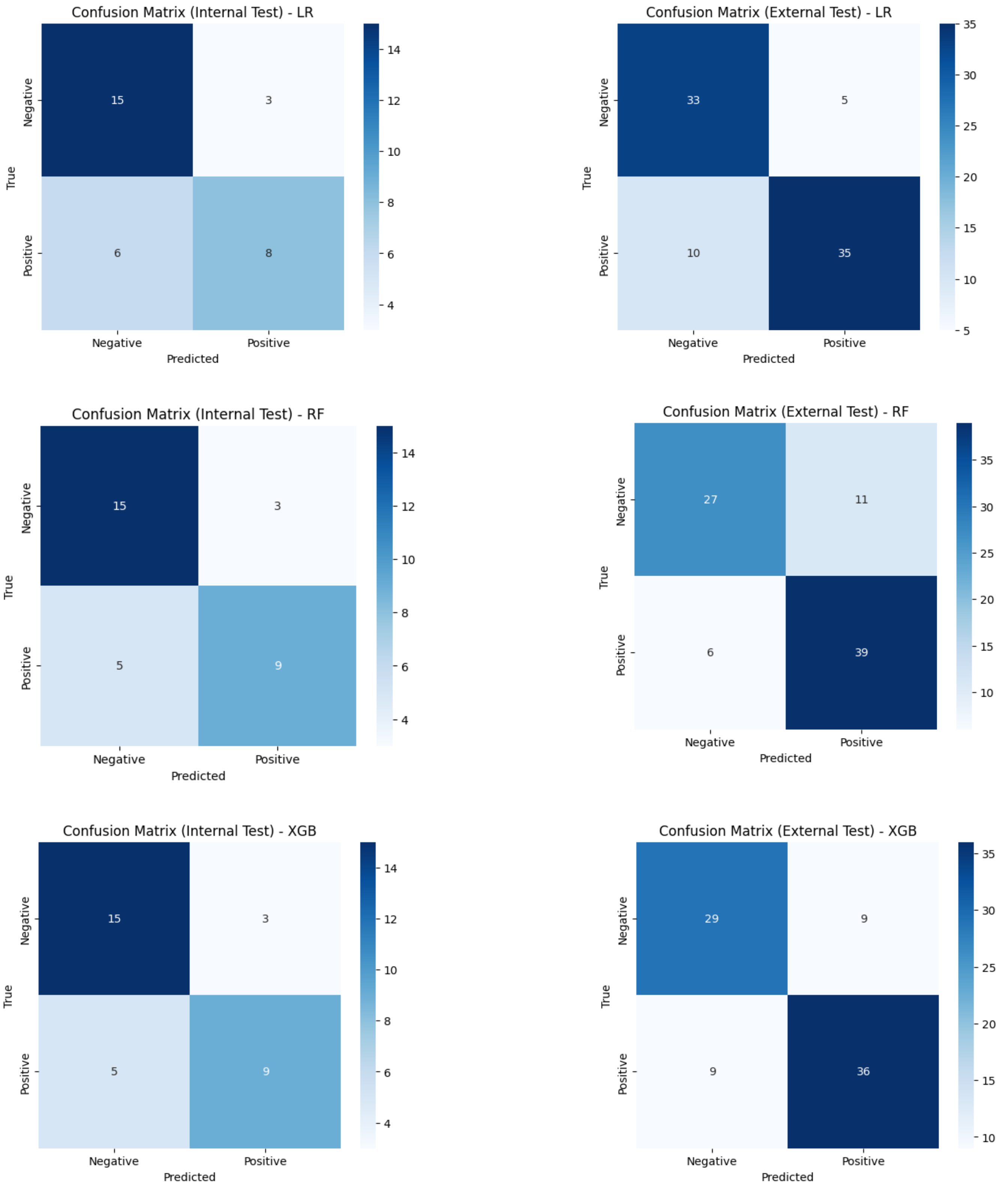

Figure 1. Confusion Matrix for Internal and External Testing of three models: Logistic Regression, Random Forest, and XGBoost in Classification of NHL vs HL Using(Nodal + Extra-Nodal) Radiomics + Age.

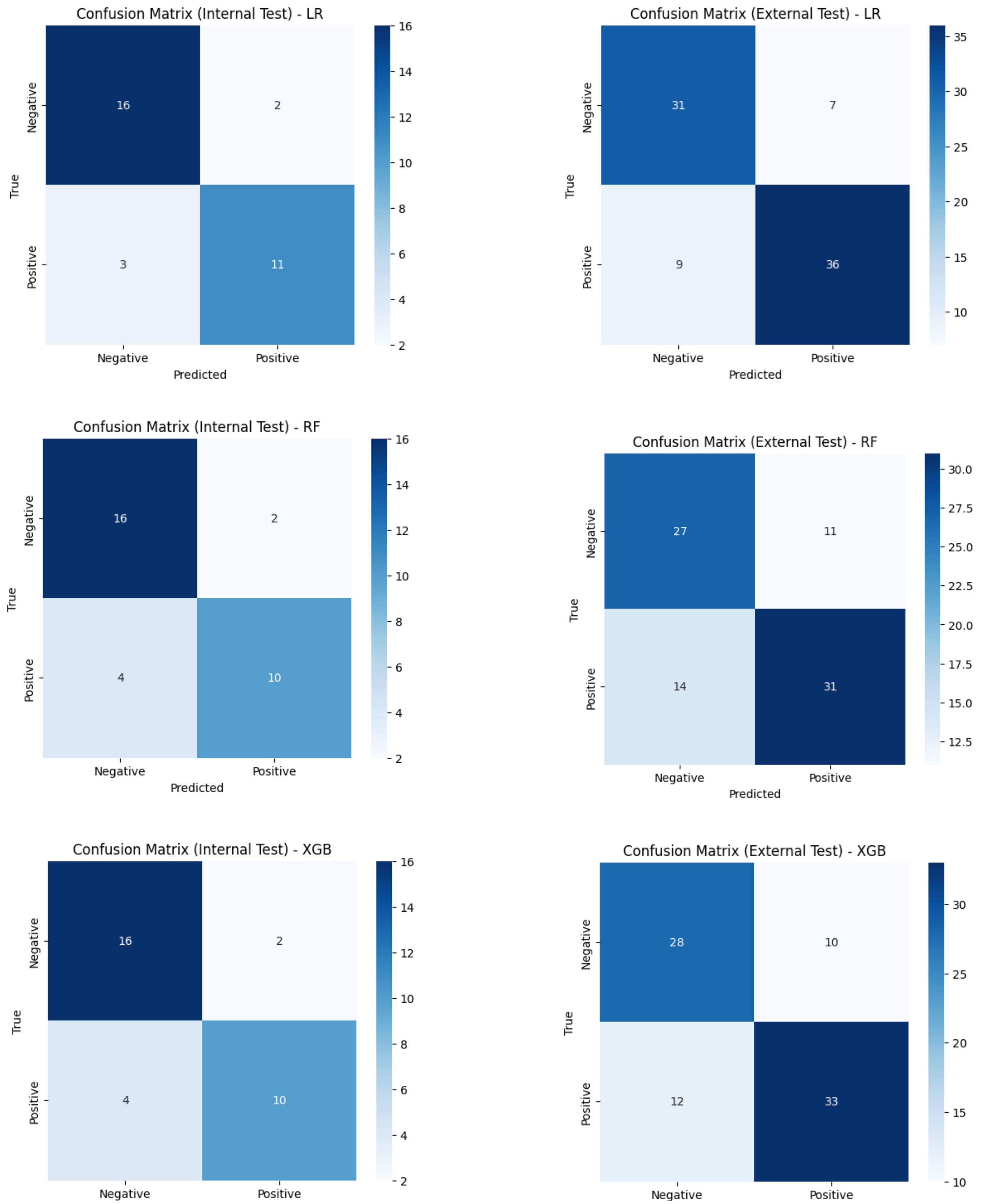

Figure 2. Confusion Matrix for Internal and External Testing of three models: Logistic Regression, Random Forest, and XGBoost in Classification of NHL vs HL Using(Nodal) Radiomics + Age.

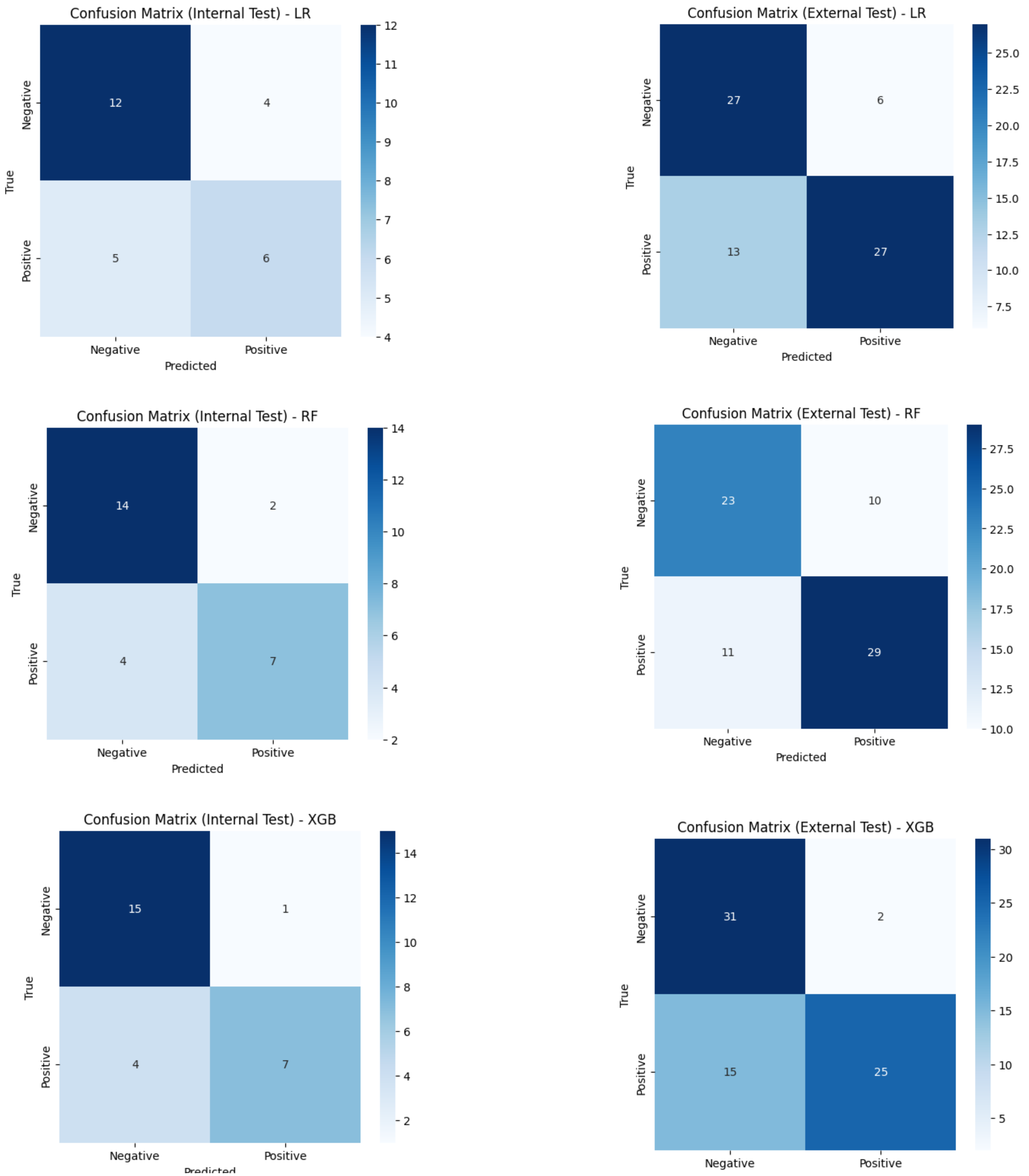

Figure 3. Confusion Matrix for Internal and External Testing of three models: Logistic Regression, Random Forest, and XGBoost in Classification of High grade non-HL vs .CHL Using(Nodal + Extra-Nodal)Radiomics + Age.

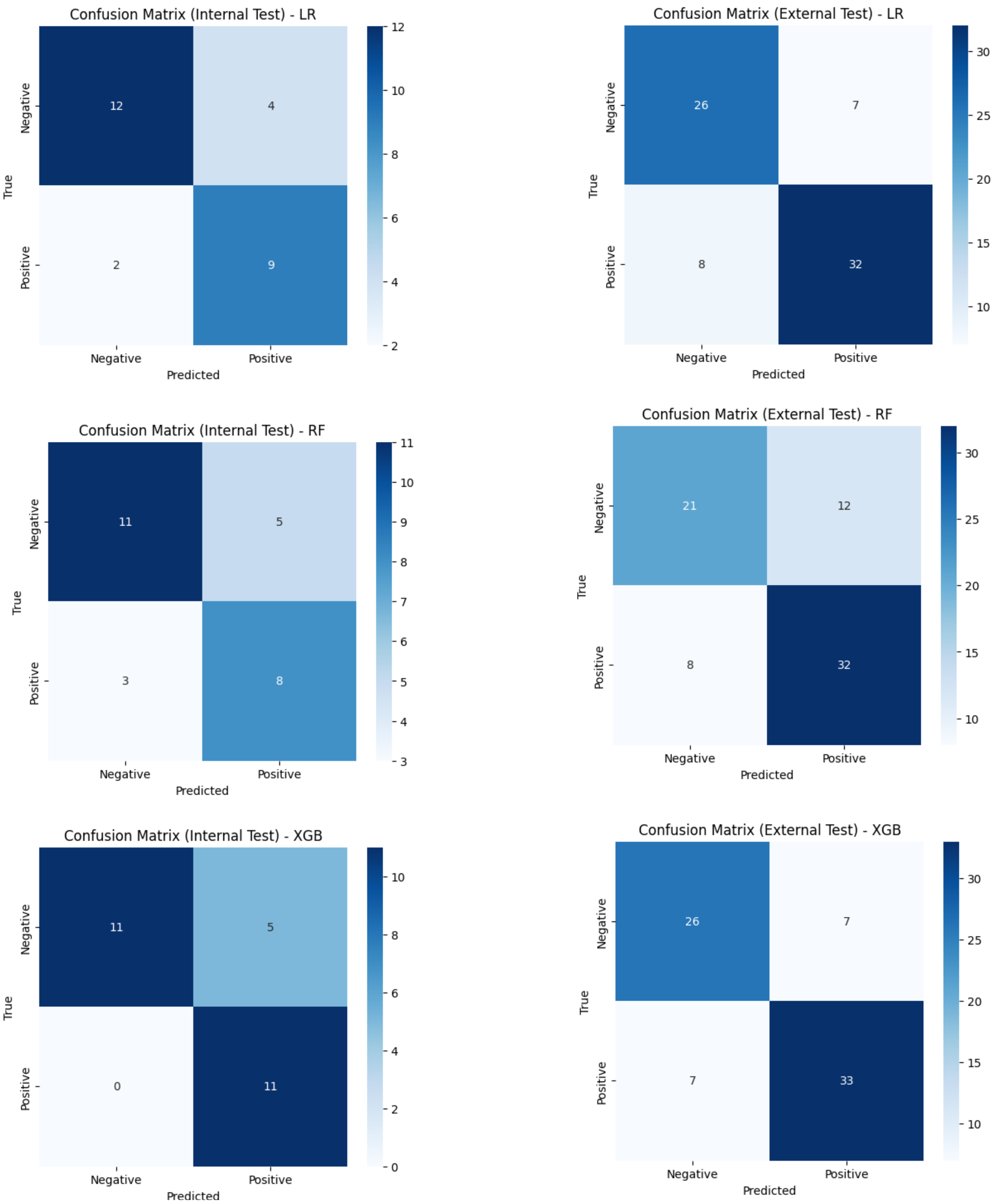

Figure 4. Confusion Matrix for Internal and External Testing of three models: Logistic Regression, Random Forest, and XGBoost in Classification of High grade non-HL vs .CHL Using(Nodal)Radiomics + Age.

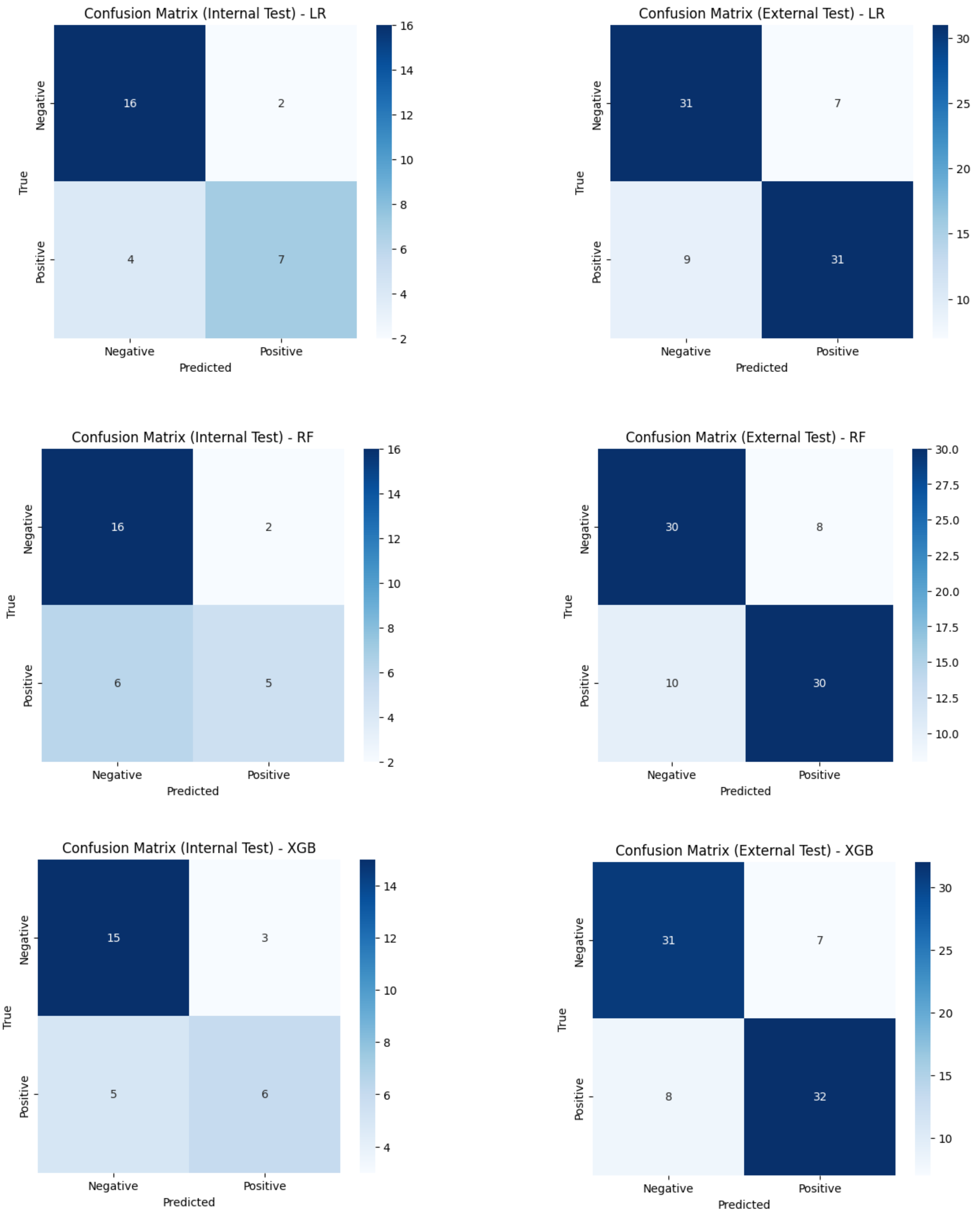

Figure 5. Confusion Matrix for Internal and External Testing of three models: Logistic Regression, Random Forest, and XGBoost in Classification of High grade non-HL vs . HL Using(Nodal + Extra-Nodal)Radiomics + Age.

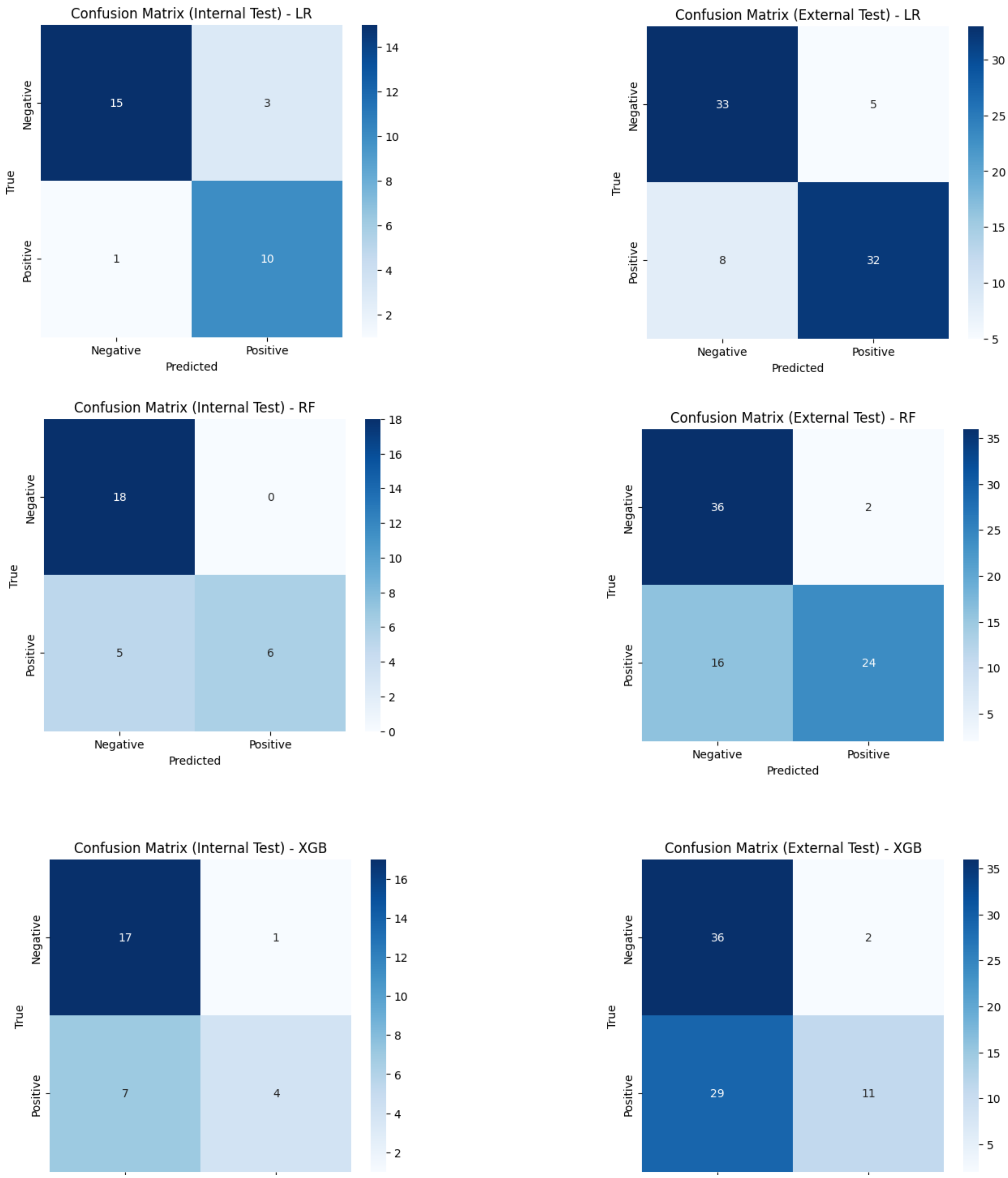

Figure 6. Confusion Matrix for Internal and External Testing of three models: Logistic Regression, Random Forest, and XGBoost in Classification of High grade non-HL vs . HL Using(Nodal)Radiomics + Age.

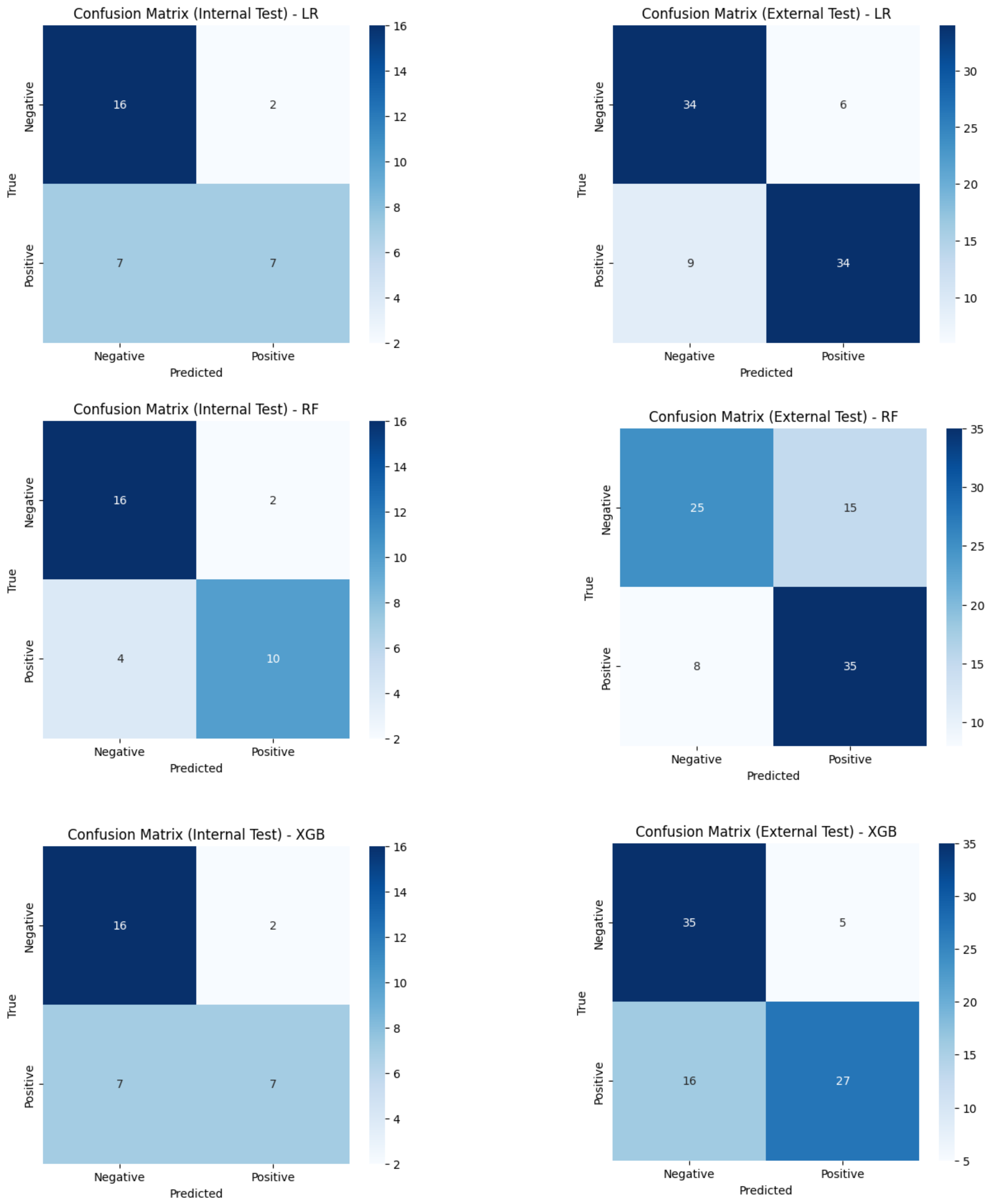

Figure 7. Confusion Matrix for Internal and External Testing of three models: Logistic Regression, Random Forest, and XGBoost in Classification of B cell vs others Using(Nodal + Extra-Nodal) Radiomics + Age.

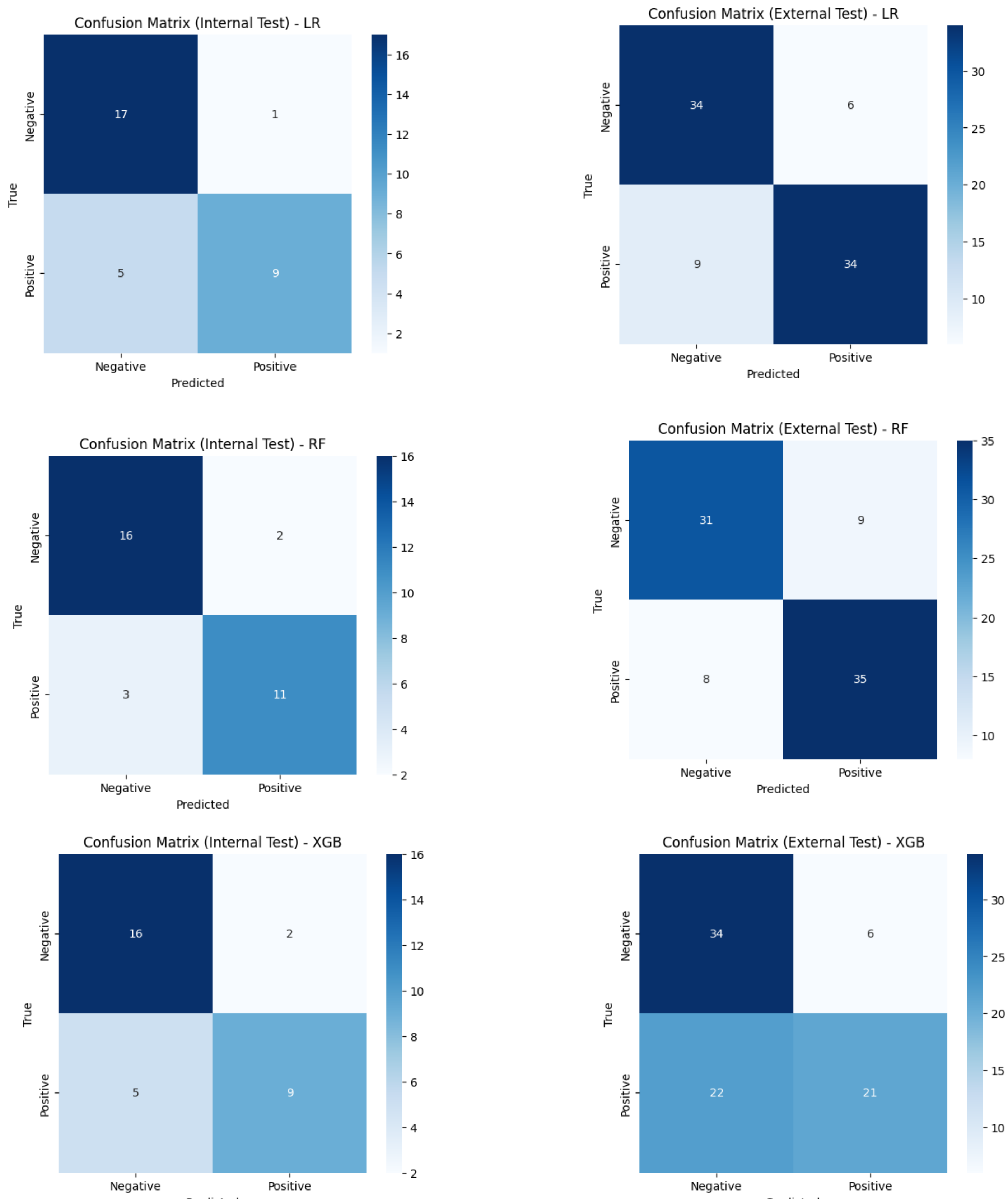

Figure 8. Confusion Matrix for Internal and External Testing of three models: Logistic Regression, Random Forest, and XGBoost in Classification of B cell vs others Using(Nodal) Radiomics + Age.

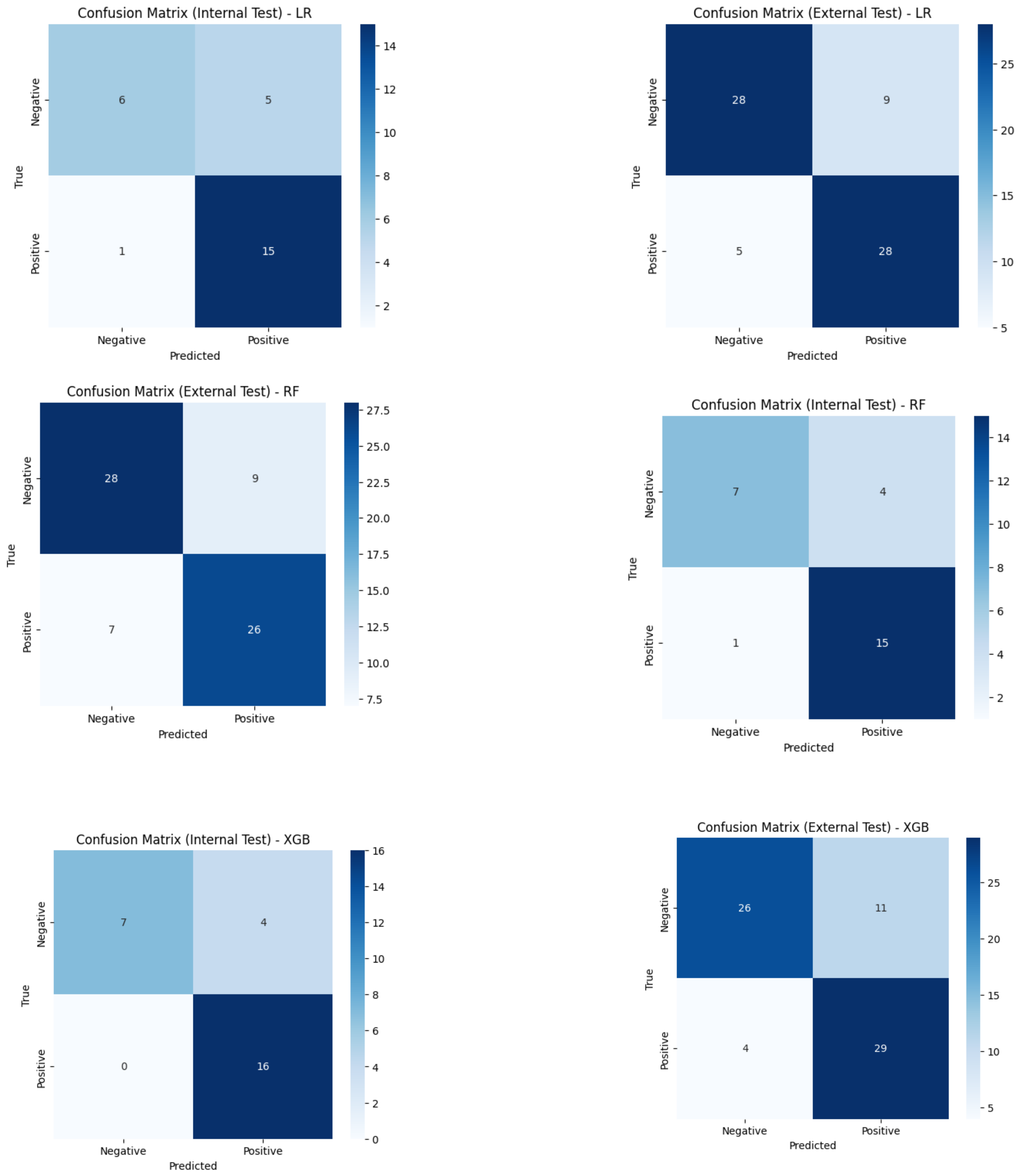

Figure 9. Confusion Matrix for Internal and External Testing of three models: Logistic Regression, Random Forest, and XGBoost in Classification of AVBD Vs. R_CHOP Candidate Using(Nodal + Extra-Nodal) Radiomics + Age.

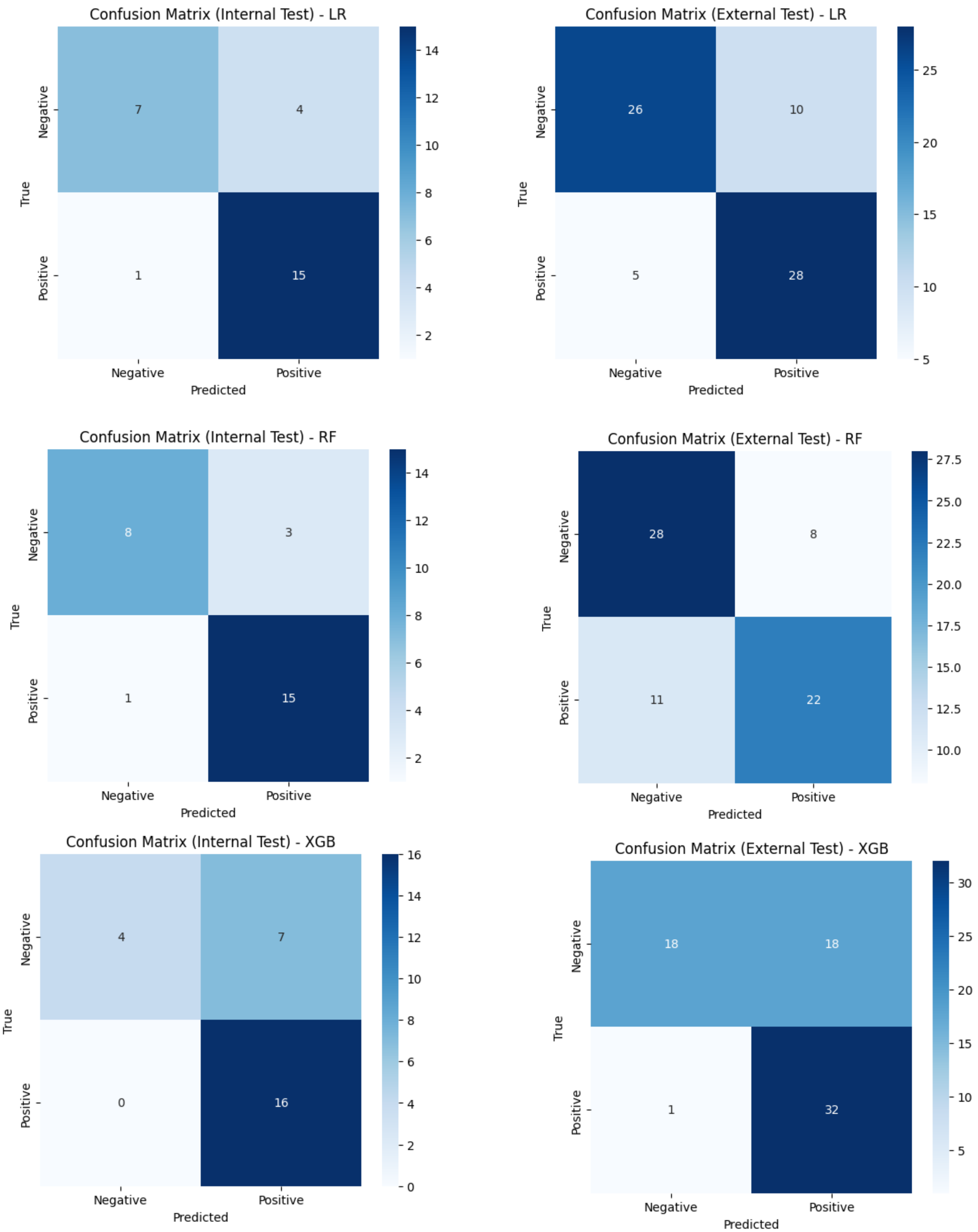

Figure 10. Confusion Matrix for Internal and External Testing of three models: Logistic Regression, Random Forest, and XGBoost in Classification of AVBD Vs. R_CHOP Candidate Using(Nodal) Radiomics + Age.

A

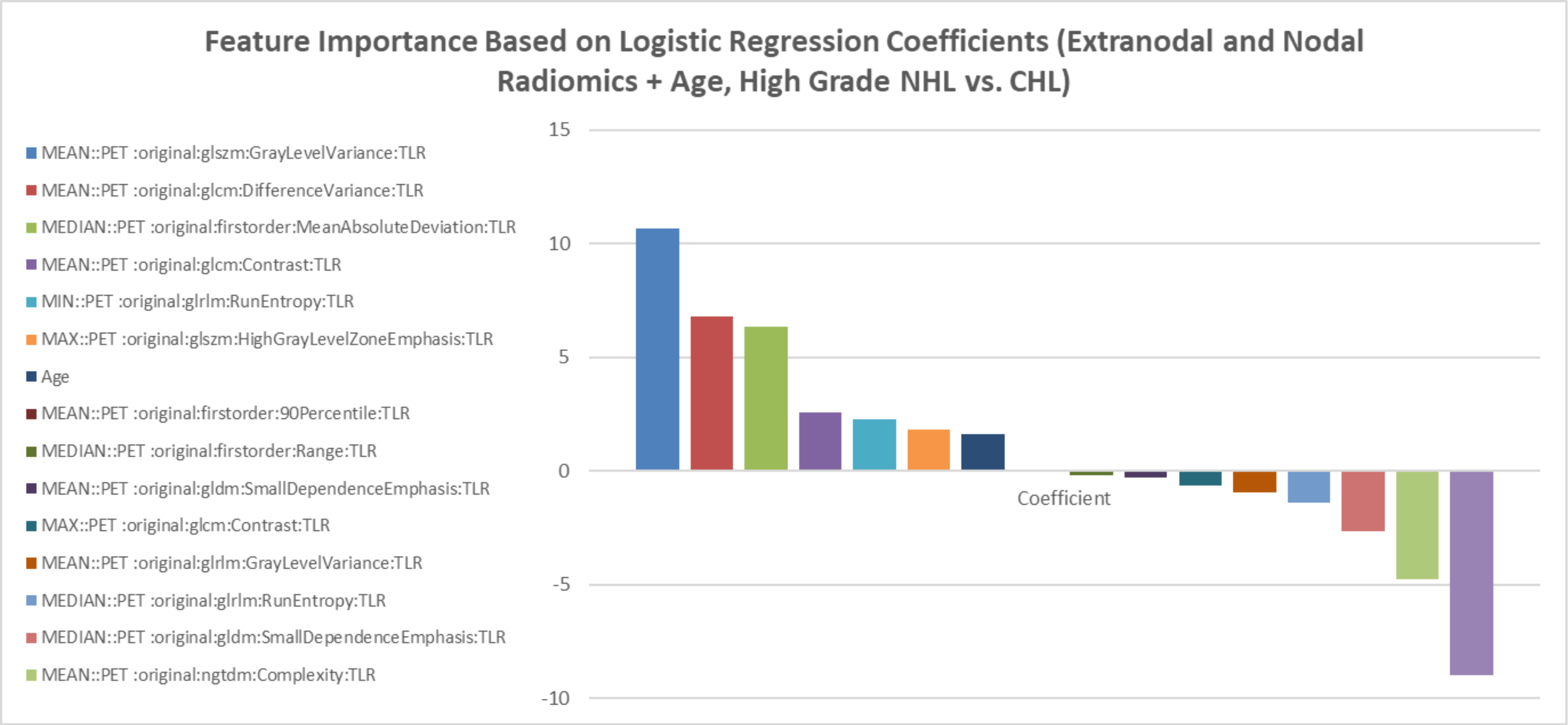

Feature Importance Based on Logistic Regression Coefficients (Extranodal and Nodal Radiomics + Age, High Grade NHL vs. CHL)
MEAN::PET :original:glszm:GrayLevelVariance:TLR
MEAN::PET :original:glcm:DifferenceVariance:TLR
MEDIAN::PET :original:firstorder:MeanAbsoluteDeviation:TLR
MEAN::PET :original:glcm:Contrast:TLR
MIN::PET :original:glrlm:RunEntropy:TLR
MAX::PET :original:glszm:HighGrayLevelZoneEmphasis:TLR
Age
MEAN::PET :original:firstorder:90Percentile:TLR
MEDIAN::PET :original:firstorder:Range:TLR
MEAN::PET :original:gldm:SmallDependenceEmphasis:TLR
MAX::PET :original:glcm:Contrast:TLR
MEAN::PET :original:glrlm:GrayLevelVariance:TLR
MEDIAN::PET :original:glrlm:RunEntropy:TLR
MEDIAN::PET :original:gldm:SmallDependenceEmphasis:TLR
MEAN::PET :original:ngtdm:Complexity:TLR
15
10
5
0
-5
-10
Coefficient

B

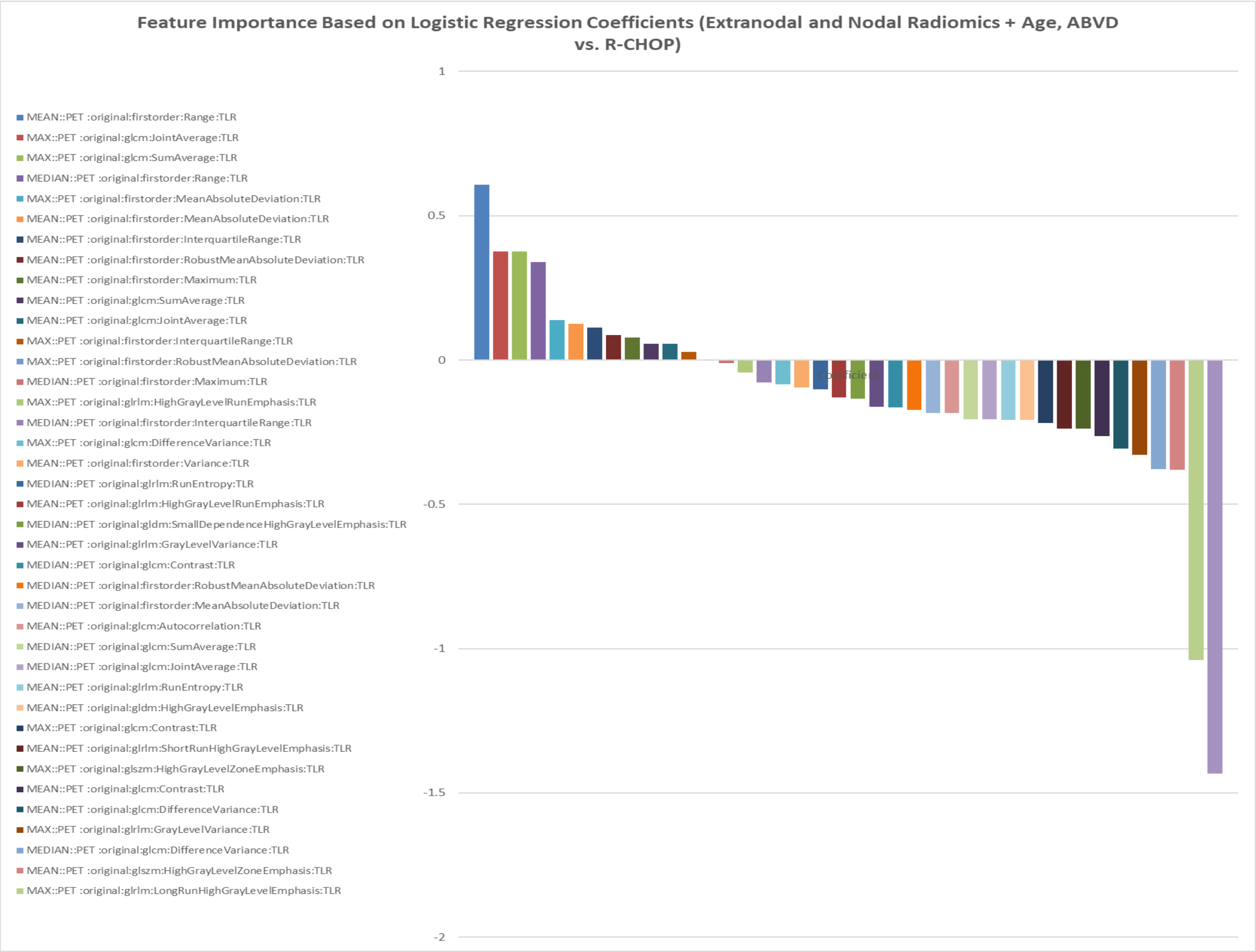

Feature Importance Based on Logistic Regression Coefficients (Extranodal and Nodal Radiomics + Age, ABVD vs. R-CHOP)
1
0.5
0
-0.5
-1
-1.5
-2
MEAN::PET :original:firstorder:Range:TLR
MAX::PET :original:glcm:JointAverage:TLR
MAX::PET :original:glcm:SumAverage:TLR
MEDIAN::PET :original:firstorder:Range:TLR
MAX::PET :original:firstorder:MeanAbsoluteDeviation:TLR
MEAN::PET :original:firstorder:MeanAbsoluteDeviation:TLR
MEAN::PET :original:firstorder:InterquartileRange:TLR
MEAN::PET :original:firstorder:RobustMeanAbsoluteDeviation:TLR
MEAN::PET :original:firstorder:Maximum:TLR
MEAN::PET :original:glcm:SumAverage:TLR
MEAN::PET :original:glcm:JointAverage:TLR
MAX::PET :original:firstorder:InterquartileRange:TLR
MAX::PET :original:firstorder:RobustMeanAbsoluteDeviation:TLR
MEDIAN::PET :original:firstorder:Maximum:TLR
MAX::PET :original:glrlm:HighGrayLevelRunEmphasis:TLR
MEDIAN::PET :original:firstorder:InterquartileRange:TLR
MAX::PET :original:glcm:DifferenceVariance:TLR
MEAN::PET :original:firstorder:Variance:TLR
MEDIAN::PET :original:glrlm:RunEntropy:TLR
MEAN::PET :original:glrlm:HighGrayLevelRunEmphasis:TLR
MEDIAN::PET :original:gldm:SmallDependenceHighGrayLevelEmphasis:TLR
MEAN::PET :original:glrlm:GrayLevelVariance:TLR
MEDIAN::PET :original:glcm:Contrast:TLR
MEDIAN::PET :original:firstorder:RobustMeanAbsoluteDeviation:TLR
MEDIAN::PET :original:firstorder:MeanAbsoluteDeviation:TLR
MEAN::PET :original:glcm:Autocorrelation:TLR
MEDIAN::PET :original:glcm:SumAverage:TLR
MEDIAN::PET :original:glcm:JointAverage:TLR
MEAN::PET :original:glrlm:RunEntropy:TLR
MEAN::PET :original:gldm:HighGrayLevelEmphasis:TLR
MAX::PET :original:glcm:Contrast:TLR
MEAN::PET :original:glrlm:ShortRunHighGrayLevelEmphasis:TLR
MAX::PET :original:glszm:HighGrayLevelZoneEmphasis:TLR
MEAN::PET :original:glcm:Contrast:TLR
MEAN::PET :original:glcm:DifferenceVariance:TLR
MAX::PET :original:glrlm:GrayLevelVariance:TLR
MEDIAN::PET :original:glcm:DifferenceVariance:TLR
MEAN::PET :original:glszm:HighGrayLevelZoneEmphasis:TLR
MAX::PET :original:glrlm:LongRunHighGrayLevelEmphasis:TLR

C

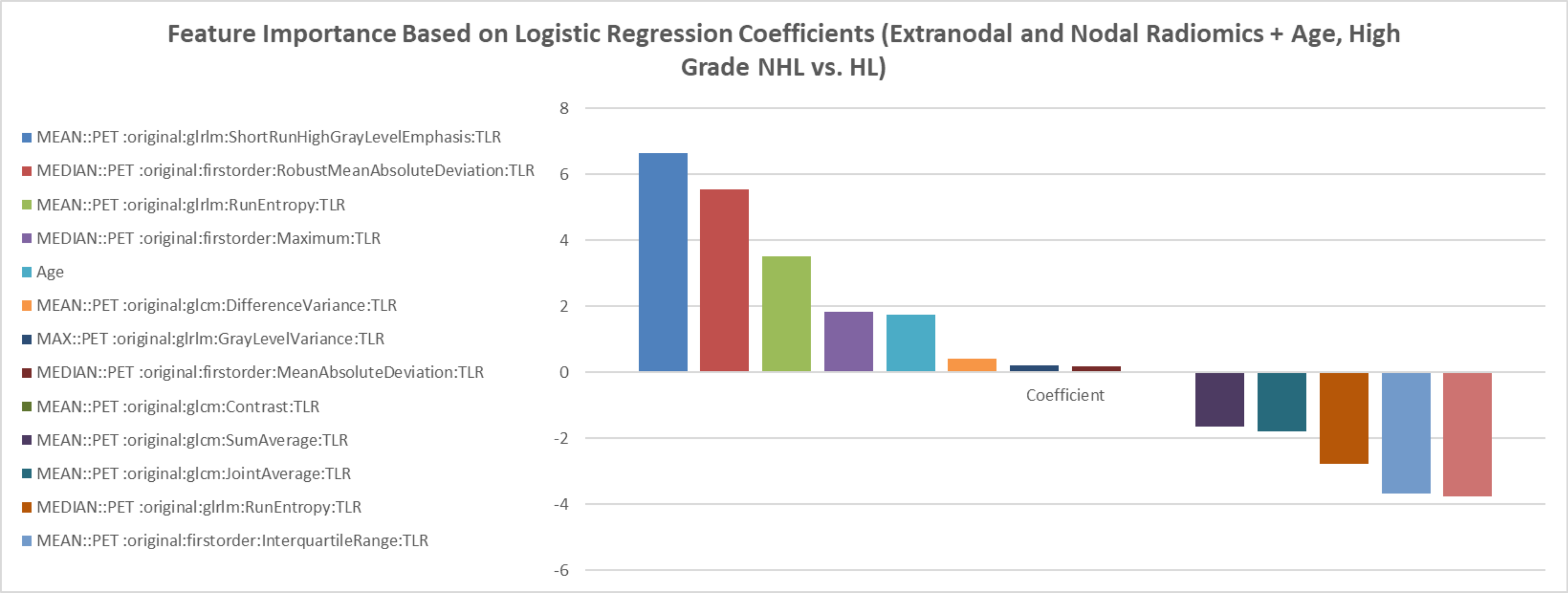
Feature Importance Based on Logistic Regression Coefficients (Extranodal and Nodal Radiomics + Age, High Grade NHL vs. HL)
MEAN::PET :original:glrlm:ShortRunHighGrayLevelEmphasis:TLR
MEDIAN::PET :original:firstorder:RobustMeanAbsoluteDeviation:TLR
MEAN::PET :original:glrlm:RunEntropy:TLR
MEDIAN::PET :original:firstorder:Maximum:TLR
Age
MEAN::PET :original:glcm:DifferenceVariance:TLR
MAX::PET :original:glrlm:GrayLevelVariance:TLR
MEDIAN::PET :original:firstorder:MeanAbsoluteDeviation:TLR
MEAN::PET :original:glcm:Contrast:TLR
MEAN::PET :original:glcm:SumAverage:TLR
MEAN::PET :original:glcm:JointAverage:TLR
MEDIAN::PET :original:glrlm:RunEntropy:TLR
MEAN::PET :original:firstorder:InterquartileRange:TLR
8
6
4
2
0
-2
-4
-6
Coefficient

D

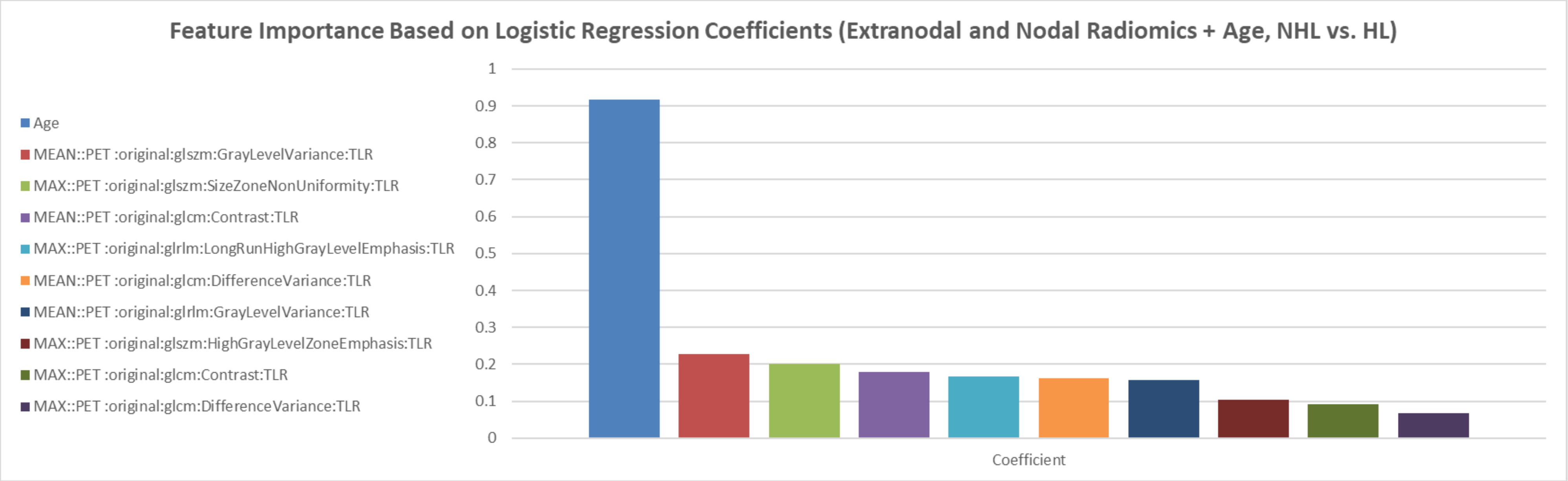
Feature Importance Based on Logistic Regression Coefficients (Extranodal and Nodal Radiomics + Age, NHL vs. HL)
Age
MEAN::PET :original:glszm:GrayLevelVariance:TLR
MAX::PET :original:glszm:SizeZoneNonUniformity:TLR
MEAN::PET :original:glcm:Contrast:TLR
MAX::PET :original:glrlm:LongRunHighGrayLevelEmphasis:TLR
MEAN::PET :original:glcm:DifferenceVariance:TLR
MEAN::PET :original:glrlm:GrayLevelVariance:TLR
MAX::PET :original:glszm:HighGrayLevelZoneEmphasis:TLR
MAX::PET :original:glcm:Contrast:TLR
MAX::PET :original:glcm:DifferenceVariance:TLR
1
0.9
0.8
0.7
0.6
0.5
0.4
0.3
0.2
0.1
0
Coefficient

E

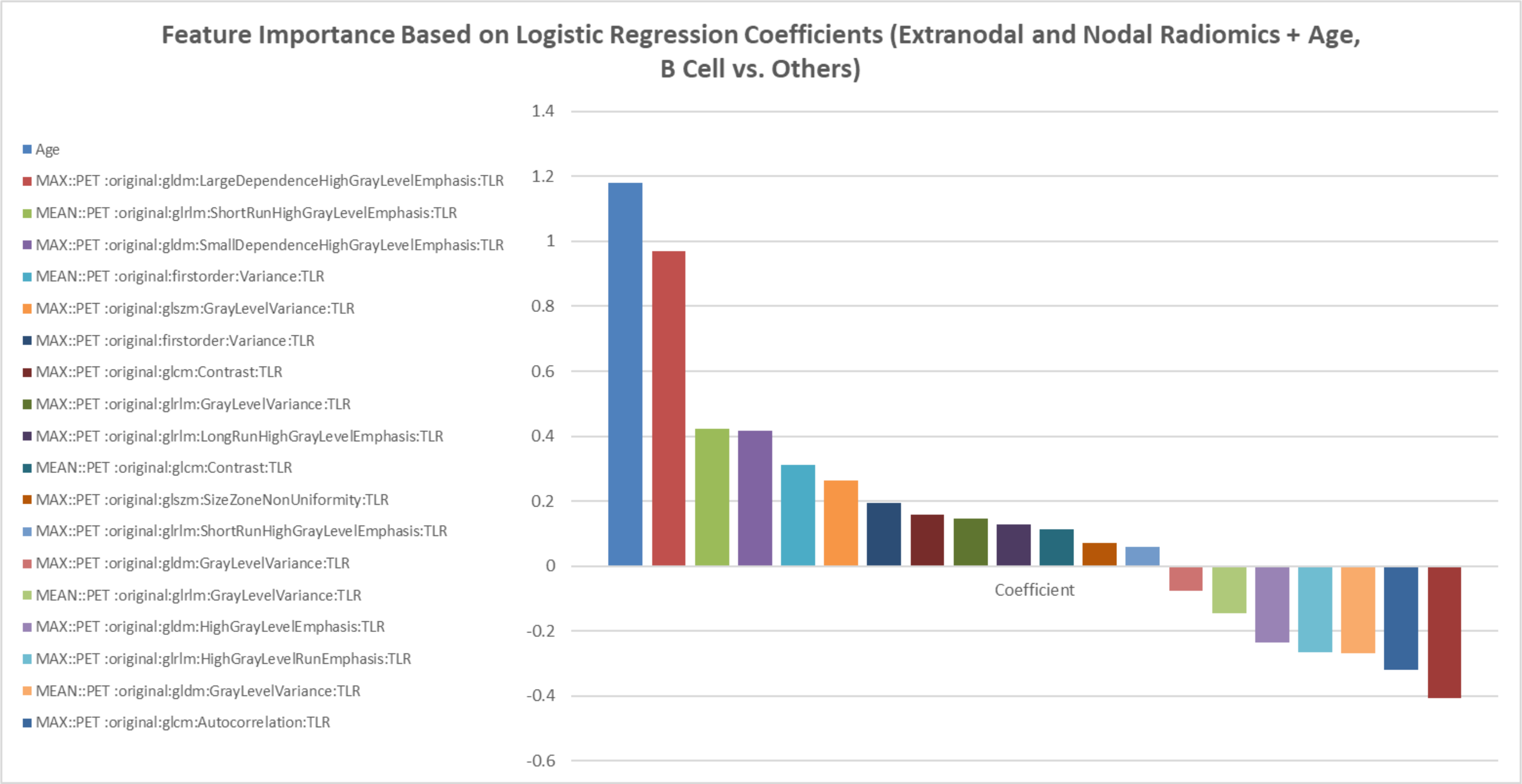

Figure 11. Feature importance based on logistic regression coefficients using extranodal and nodal radiomics features plus age: (A) High-grade NHL vs. CHL, (B) ABVD vs. R-CHOP, (C) High-grade NHL vs. HL, (D) NHL vs. HL, (E) B-cell lymphoma vs. others.
